# Nothingness Is All There Is: An Exploration of Objectless Awareness During Sleep

**DOI:** 10.3389/fpsyg.2022.901031

**Published:** 2022-06-10

**Authors:** Adriana Alcaraz-Sánchez, Ema Demšar, Teresa Campillo-Ferrer, Susana Gabriela Torres-Platas

**Affiliations:** ^1^Department of Philosophy, Centre for the Study of the Perceptual Experience, University of Glasgow, Glasgow, United Kingdom; ^2^Monash Centre for Consciousness and Contemplative Studies, Department of Philosophy, Monash University, Melbourne, VIC, Australia; ^3^Ruhr University Bochum, Bochum, Germany; ^4^Donders Institute for Brain, Cognition and Behaviour, Radboud University Medical Centre, Nijmegen, Netherlands; ^5^Geri-PARTy Research Group, Jewish General Hospital/Lady Davis Institute, McGill University, Montreal, QC, Canada

**Keywords:** dreamless sleep experiences, witnessing-sleep, objectless awareness, microphenomenology, qualitative research

## Abstract

Recent years have seen a heightened focus on the study of minimal forms of awareness during sleep to advance the study of consciousness and understand what makes a state conscious. This focus draws on an increased interest in anecdotical descriptions made by classic Indian philosophical traditions about unusual forms of awareness during sleep. For instance, in the so-called state of witnessing-sleep or luminosity sleep, one is said to reach a state that goes beyond ordinary dreaming and abide in a state of just awareness, a state in which one is not aware of anything else other than one’s own awareness. Moreover, for these traditions, this state is taken to be the essence or background of consciousness. Reports on such a state opens the door to exciting new lines of research in the study of consciousness, such as inquiry into the so-called objectless awareness during sleep—states of awareness that lack an ordinary object of awareness. In this two-staged research project, we attempted to find the phenomenological blueprints of such forms of awareness during sleep in 18 participants by conducting phenomenological interviews, informed by a novel tool in qualitative research, the micro-phenomenological interview (MPI) method. Following a phenomenological analysis, we isolated a similar phase across 12 reported experiences labeled as “nothingness phase” since it described what participants took to be an experience of “nothingness.” This common phase was characterized by minimal sense of self—a bodiless self, yet experienced as being “somewhere”—, the presence of non-modal sensations, relatively pleasant emotions, an absence of visual experience, wide and unfocused attention, and an awareness of the state as it unfolded.

## Introduction

The investigation of the variety of conscious experiences during sleep has recently received growing attention for the study of the nature of consciousness. In particular, recent years have seen an increased interest in the investigation of minimal forms of consciousness during sleep reported in contemplative traditions (see Thompson, 2014, 2015; Windt, 2015, 2020; Windt et al., 2016). Such forms of consciousness have been widely reported for centuries in different Indian philosophical traditions, such as the Advaita Vedānta and some lineages in Indo-Tibetan Buddhism. For instance, in the Vedas, we find mentions to the state of “*sushupti*,” known as “witnessing-sleep,” taken by the Advaita Vedānta and Yoga schools as a special state of consciousness, distinct from waking and dreaming.^1^ During sushupti, we are said to lack the sort of object-directed awareness experienced during ordinary wakefulness or dreaming; the state of sushupti is said to lack any sort of cognition or perception (see Olivelle, 1998). Thus, the state of sushupti can only be known or accessed when one is entering or emerging from it. Nevertheless, these schools take sushupti as a state of phenomenal consciousness—a state in which there is something it is like to be in it, an experiential state (see Nikhilananda, 1949; Thompson, 2014, 2015). Some authors understand such a state as a state of non-dual awareness—a state of awareness that does not involve the distinction between the experiencer, an “I,” and an object of awareness, a state of self-luminous awareness (Prasad, 2000; Sharma, 2001). Moreover, these schools take sushupti to be an instance of “pure awareness,” a state of consciousness-as-such (see Metzinger, 2019 for a discussion).

Similarly, in the Dzogchen tradition in Indo-Tibetan Buddhism, we find mentions to what is regarded as the state of “clear light” or “luminosity,” a state that can be reached during dreamless sleep through highly skilled meditative practices, such as Yoga Nidra or luminosity yoga (Norbu, 1983; Wangyal, 1998). In such a state, we are said to encounter the nature or essence of the mind, to realize the true nature of consciousness (cf. Ponlop, 2006). Accounts of such a state of objectless awareness during sleep have brought up the notion of “lucid dreamless sleep,” a state of meta-awareness during sleep in absence of dream content (see Thompson, 2014, 2015; Windt, 2015; Windt et al., 2016). Anecdotical reports of this state of minimal consciousness during sleep open the door to exciting lines of research in consciousness studies. For instance, Windt (2015) has proposed that some forms of objectless awareness during sleep could be an instance of the minimal phenomenal experience—the simplest form of consciousness one can have. Metzinger (2020) has followed on this and investigated further the common denominator between different forms of experience that could be said to be minimally conscious, including those had during meditation, to find the phenomenological structure of consciousness-as-such.

Notwithstanding the descriptions of objectless conscious sleep found in contemplative traditions, the phenomenological blueprints of such an experience are still unclear. First, most descriptions usually rely on anecdotical reports and are rarely based on first-hand experiences. Second, these descriptions tend to be provided by individuals embedded in a specific belief system (see Alexander, 1990; Mason et al., 1990, 1997; Travis, 1994; Travis and Pearson, 2000; Mason and Orme-Johnson, 2010). Third, due to its nature, an experience that is said to be “objectless” and lacks a subject-object distinction is extremely difficult to report and to characterize and, thus, presents great challenges for how it could be studied empirically (Alcaraz-Sanchez, 2021). Similarly, there is the question as to whether such reports are indeed an instance of “objectless” awareness or do indeed involve some sort of object-directed awareness.

Despite the described challenges, theoretical accounts establishing what may count as an instance of objectless awareness require a phenomenological clarification of such experiences. If one was to experience an awareness lacking a distinct object of awareness during sleep, what would it look like? Recent empirical and conceptual work has tried to shed light on this question by investigating reports of objectless awareness in expert meditators (cf. Gamma and Metzinger, 2021), or by focusing on reports of this phenomenon had during sleep (cf. Alcaraz-Sanchez, 2021). The present research project aimed at furthering the investigation of this phenomenon by exploring in more detail the phenomenology of possible instances of “objectless” awareness during sleep. The project involved two phases. The first phase consisted of the distribution of an online survey that asked participants about different forms of sleep phenomena they might have experienced within the last month, including forms of awareness that could be taken to be “objectless” or “contentless” (lacking a clear content of awareness; Alcaraz-Sánchez, *under preparation*). The second phase, focus of the present paper, involved phenomenological interviews with participants shortlisted from the survey.

## Materials and Methods

### Research Questions

The study aimed at shedding light on different instantiations of objectless awareness during sleep, defined here as an awareness that lacks a distinct object of awareness, by investigating systematically the phenomenology of these experiences. The goal was to explore what is taken by individuals as an experience that lacks a distinct object or content of awareness had during sleep, regardless of whether such an experience should properly be accounted as “objectless,” or whether this is the sort of state alluded to by Indian philosophical traditions as “witnessing-sleep” or “clear light sleep.” Moreover, our study left aside any considerations as to whether such an experience is the most minimal possible form of experience, or if it should be taken to be the essence of consciousness. As such, some of the research questions poised to be answered were: which sort of sleep experience do people take to be “objectless”? What are the potential markers, similarities, and descriptions of these candidate “objectless” experiences?

### Participants

The participants for this second phase of the research were selected from the “*Objectless sleep experiences online survey*” (Alcaraz-Sánchez, *under preparation*), which asked participants to answer a series of open-ended and multiple-choice questions exploring their sleep experiences, including sleep onset awareness, dream awareness, and sleep awareness lacking any other mentation. From those participants wishing to participate in the second stage of the research, we shortlisted those mentioning what they took to be an instance of objectless awareness during sleep had within the last month and well-remembered. To that aim, we considered as entry points for exploring potential instances of objectless awareness during sleep the following:^2^

- An awareness following the dream environment disappearing or dissolving
- An awareness had while sleeping in absence of any other perception or cognition
- An awareness of the process of falling asleep or waking up without another object of awareness
- A feeling of knowing that one was conscious while sleeping upon awakening without relating it to a dream experience

We shortlisted a total of 38 participants who meet the selection criteria (from 573 answering the survey), and of those, we selected 18 who were interviewed in a total of 21 interview sessions (total 34.19 h, *μ* = 1.62 h, SD = 0.81 h). From the reports provided, we selected those describing experiences that matched or approximated the definition of the targeted state of objectless awareness as indicated above. Here, we will refer to “participants” as the individuals whose experiences were selected for this paper (*n* = 12, *μ* = 36.5, seven males and five females).^3^ A follow-up paper will cover the remaining reports and the experiences that were not included in the present analysis.

All participants signed an informed consent form to partake in the interview, and the study was approved by the Ethics Committee of the College of Arts at the University of Glasgow.

### Interview Procedure and Protocol

The interviews were carried out by the first author (AA-S) and the second author (ED) in 1:1 sessions *via* Zoom and lasted an average of 1.5 h. We adopted an interview protocol inspired by the micro-phenomenological interview (MPI) technique by Petitmengin (1999, 2006) to gather fine-grained subjective reports. The MPI technique guides the interviewee through the recollection process and helps them to focus on their subjective feelings—how they felt in a particular moment—moving them away from preconceptions and judgments about their experience.^4^ Moreover, this method aids the interviewee to uncover unnoticed aspects of their experience, which otherwise would have been difficult to assess (for a full account of the MPI protocol, see Petitmengin, 2006; Petitmengin et al., 2018). In the interview sessions, we began exploring the entirety of the reported episode, and then zooming in to those phases that were of most interest for our research question, focusing both on how the experience unfolded to a state taken to be “objectless” and the specific experiential structures involved (see Supplementary Material for examples of some interview questions and short excerpts from the interviews).

The interview sessions consisted of two parts. First, participants were asked to perform a short mental task consisting of mentally spelling a given word and were afterward interviewed about this experience of spelling. Second, they were interviewed about a recent experience of what they took to be objectless awareness during sleep. For both reports provided (about the experience of spelling the word and about their sleep experience), participants were asked afterward to rate the degree of completeness and accuracy of their reporting, the vividness of the recalled experience while they were being interviewed, as well as the extent to which they felt they might have invented or fabricated some elements of the description, and the ease (or difficulty) of articulating the experience within the interview.

### Qualitative Analysis

We undertook a phenomenological analysis of the interviews verbatim using a combination of tools by the MPI method (Petitmengin et al., 2018; Valenzuela-Moguillansky and Vásquez-Rosati, 2019), grounded theory (Charmaz, 2006; Thornberg and Charmaz, 2014), and thematic analysis (Braun and Clarke, 2021). The analysis procedure consisted of the following steps:

#### Initial Examination and Data Preparation

AA-S and ED undertook an initial examination of the interviews and identified patterns, structures and research questions that emerged from them. AA-S selected those sections of the verbatim relevant for the analysis; comments, judgements or evaluations made by the participants were removed, following the micro-phenomenological analysis approach (cf. Petitmengin, 2006; Petitmengin et al., 2018).

#### Thematic and Categorical Analysis

AA-S and ED started by identifying patterns in each of the interviews that described how the experience reported unfolded over time (diachronic structure; see Petitmengin, 2006; Petitmengin et al., 2018). These patterns were compared across the reports selected in several reiterations to identify similar diachronic structures as well as for isolating an experiential episode resemblant to our targeted experience of objectless awareness during sleep. From this commonly isolated phase, AA-S and ED identified different dimensions that emerged from the descriptions by considering the distinctive aspects of the phase described (synchronic structure). These dimensions were converted into categories through a process of thematic analysis by grouping those dimensions that could be classified under the same theme (see Charmaz, 2006; Valenzuela-Moguillansky and Vásquez-Rosati, 2019). Thus, each theme clustered similar descriptions by assigning a “label” that could give meaning to those descriptions and aimed at leaving outside any previous preconceptions or theoretical accounts that the researchers might have had—the “labels” are intended to work as placeholders to group together a set of descriptions.

The resulting categories were classified into three levels: first (higher-level categories), second (sub-categories), and third (sub-sub-categories). Given their level of abstraction, the first-level and second-level categories only considered those dimensions that were common for most participants, while the third-level categories specified categories that might have been present for only one participant. Thus, the most abstract categories are not meant to comprise an exhaustive categorization.

#### External Analysis

Two external researchers (TCF and SGTP) carried out another round of coding of the phenomenal descriptions by assigning a pre-established code (consisting of a category and sub-category). The external researchers were provided a list and full descriptions of the categories and sub-categories isolated in the thematic analysis (see Supplementary Material). This process was assessed through an intercoder agreement score (detailed in section Quantitative Analysis).

#### Final Analysis and Redefinition of Categories

AA-S revised the external coding, examined those categories that lead to higher disagreement amongst coders and redefined or eliminated categories if necessary.

### Quantitative Analysis

We conducted two explorative quantitative analyses to inform the adequacy of the interview protocol and the phenomenological analysis. For the first, during the interview sessions, we asked participants to provide a self-rating of the degree of vividness, completeness, articulation, and accuracy of the recollection of the experience (“0” meaning very low and “10” very high), as well as the extent to which they felt they might have invented or fabricated some of the elements of the description (“0” meaning none and “10” meaning all). They self-rated both, the recollection of the experience of spelling and the potential experience of objectless sleep, explored, respectively, in the first and second part of the interview session. We calculated the means for the scores provided for each dimension and each condition (see section Individual Self-Ratings).

For the second explorative analysis, we run Fleiss’ Kappa to determine the degree of agreement between the three coders (the two external researchers, and the main author) on their classification of the different categories isolated in the thematic analysis. We also run Fleiss’ Kappa for each combination of categories and subcategories to explore which categories had a higher intercoder agreement across coders for the phase of interest in the analysis (see section Intercoder Agreement).

The exploration of the self-ratings and the intercoder agreement was done using the open-access R studio for statistical computing (R Core Team, 2021).

## Results

### Phenomenological Analysis

#### Diachronic Structure

**Figure 1:**
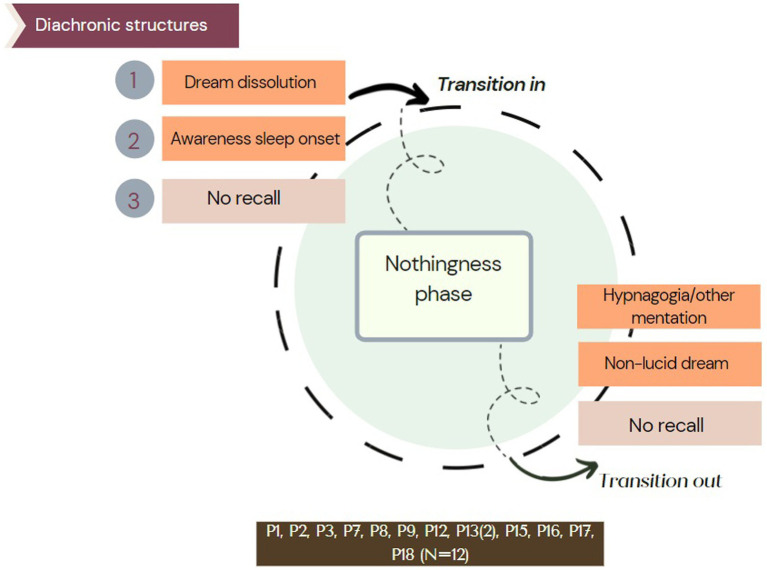
Common phase isolated in the reports. This common phase was a sub-phase of their overall described experience, labeled as “nothingness phase” in the analysis.

From the first step of the thematic and categorical analysis, we identified a common experiential phase across participants labeled as “nothingness phase” and identified three different “diachronic structures” (see Figure 1) across the reports.

##### Diachronic Structure 1: State of Void Following Dream Dissolution

For participants matching this structure (P1, P2, P15, and P18), the targeted episode preceded a lucid dream where they felt very immersed in, with a sense of being able to control the unfolding events. The lucid dream description was merely used during the interview to aid the recollection process and was not fully explored. This first structure was characterized by the dream scenery dissolving and completely disappearing. For three of the participants, the dissolution was triggered by something they actively did in the dream, such as adopting a meditation posture (P2), jumping in the air (P15), or shouting to another dream character (P18). Except for P2, who actively sought to dissolve the dream, the other two did not consciously intend this to happen. P1 described how the dissolution was also unintended, in this case, following an explosion in their dream.

**Figure 2:**
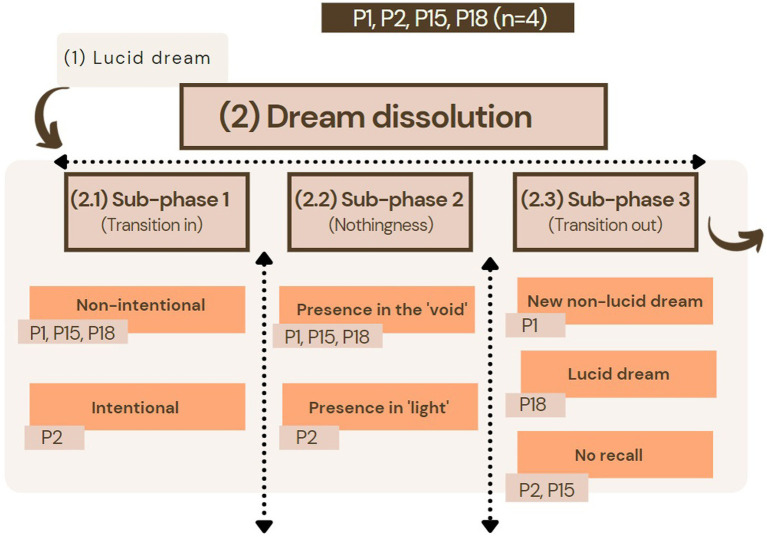
Diachronic structure 1 was characterized by the experience of the dissolution of a lucid dream preceding the sub-phase “nothingness” in participants P1, P2, P15, and P18 (*n* = 4). This sub-phase can be reached intentionally or non-intentionally.

The “nothingness phase” following the dream’s dissolution unfolded differently for each participant (see Figure 2). P1, P15, and P18 reported moving to a phase in which, while remaining aware, they said to lack any bodily sensations or imagery. For P2, the dissolution unfolded to an episode where they said to have lost a sense of being “themselves” in the experience, yet they identified themselves with a “light.” After the “nothingness phase,” both P1 and P18 mentioned moving to a different non-lucid dream. P1 described how a “blue light” came and “shake(d) them up” and, suddenly, they were transported to a new dream scenery. P18 actively sought to “recover” their dream scenery by looking for an element that was present in their previous lucid dream. P2 and P15 said not to remember what came after.

##### Diachronic Structure 2: State of Nothingness Following Sleep Onset Awareness

**Figure 3:**
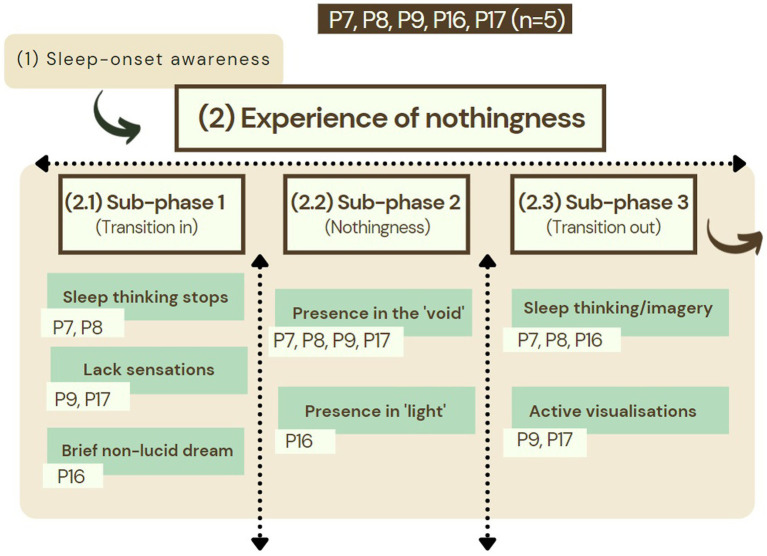
Diachronic structure 2 was characterized by a transition into the void or nothingness without a preceding lucid dream dissolution in participants P7, P8, P9, P16, and P17 (*n* = 5). Instead, for these participants there was an awareness of their sleep onset and perception of brief hypnagogic/dreaming imagery, noticing their thoughts stopping, or the absence of bodily sensations.

For five participants (P7, P8, P9, P16, and P17), the “nothingness phase” did not follow a dream’s disappearance, but instead occurred after a period of awareness during the process of falling asleep. For P16 there was a distinctive bodily feeling while falling asleep, as well as some brief non-lucid dream imagery which resulted in a state where they felt as being “bathing in light.” For the other four, there was a realization of thoughts stopping (P7), the lack of bodily feelings (P9 and P17), or the lack of any feelings at all (P8), after engaging in some form of relaxation technique while falling asleep. What followed was a phase characterized as the “void” (P8, P9, and P17), “nothingness” (P7 and P9), or “only light” (P16). This “nothingness phase” terminated in a more heterogeneous manner than diachronic structure 1 (see Figure 3). Both P7 and P8 said to become aware again of their thoughts, while P7 transitioned to a state in which they felt their body distorted accompanied with a feeling of being in bed. P8 reported that, during this episode of being in the “void,” they realized to be having the sort of experience they wanted to discuss in their upcoming interview, and slowly recovered their bodily sensations, including a feeling of being in bed. Both P9 and P16 transitioned to a different phase, in which they chose to actively visualize imagery. P9 took the opportunity to execute what they regarded as “experiments” to see whether they could send “their energy” to a relative of theirs by imagining this energy traveling from the location of their sleeping body to a relative’s home. P16 visualized a series of colors and geometric forms hoovering above their head, followed by an increasing awareness of their bodily sensations. P17, who reported experiencing the “void” frequently, described not being able to remember what happened after the “void” phase this time, but mentioned other instances during which they took advantage of this experience to initiate a lucid dream under their will in the past.

##### Diachronic Structure 3: Sudden Awareness of the State of Nothingness, No Previous Memory

For the remaining three participants (P3, P12, and P13), there was not a recall of what preceded the “nothingness phase.” Instead, they said to have just “bec[o]me aware” of “nothing” while sleeping. In the case of P3, they remembered undertaking a relaxation technique while falling asleep, paying attention to their bodily feelings, but were not able to recall what happened afterward. All they remembered was that, suddenly, there was a “tapping” which was not felt as a bodily sensation, but it “felt like the tapping itself felt itself,” an experience they said lacked an “explicit sense of self.” P12 said that other nights they have been aware of transitioning from what they called the “black spot” to this “nothingness phase.” However, in the case of the particular reported experience, they said to have just become aware of a “dark spot” taking over the experience, without recollection of what came before. Then, they transitioned into a non-lucid dream. P13 described the “nothingness phase” as a “very intense state,” where sound was all that there was, after which they became aware of their breath and were able to engage in a meditative practice.

#### Synchronic Categories

This section introduces the different experiential dimensions isolated only for the phase of the report labeled as the “nothingness phase” (see Supplementary Material for a full list of dimensions across the selected reports).

##### Sense of Self

One of the most salient features of the common “nothingness phase” across the 12 participants were the numerous descriptions alluding to how participants felt “themselves” within the experience or how they took the experience to be their own. Since varied aspects of the experience of sense of self were described, we isolated four sub-categories: “**1A-Bodily ownership**,” “**1B-Spatial self-location**,” “**1C-Perspective**,” and “**1D-Agency**.” Each sub-category was, in turn, broken down into third-level categories (see Table 1). While these categories aim to describe the distinctive features of each description, they are not mutually exclusive, and one single description can pertain to more than one second- and third-level category.

**Table 1:** Second- and third-level categories and the number of participants mentioning them for the first-level category “***Sense of self***.”

1. Sense of self	
1A. Bodily ownership	***2- Weak embodiment/lack form*:** P13, P15, P18 (*n* = 3) ***3- Distorted:*** P16 (*n* = 1) ***4- Minimal identification:*** P1, P2, P13, P7, P8, P9, P12, P17 (*n* = 8)
1B. Spatial self-location	***1- Physical:*** P7 (*n* = 1) ***3- Indeterminate:*** P1, P9, P12, P15, P18 (*n* = 5) ***4- Minimal:*** P8, P13, P17 (*n* = 3) ***5- No clear boundaries*:** P8, P16 (*n* = 2) ***6- Absent:*** P2, P3 (*n* = 2)
1C. Perspective	***1- Regular*:** P12, P18 (*n* = 2) ***2- Fluctuating:*** P2, P18 (*n* = 2) ***3- Minimal:*** P7, P8, P15 P16, P17 (*n* = 5) ***4- Absent:*** P3 (*n* = 1) *None: P1, P9 (n = 2)*
1D. Agency and attitude	***1- Active:*** P1, P7, P13, P18 (*n* = 4) ***2- Receptive:*** P1, P7, P12, P15 (*n* = 4) ***3- Lost control:*** P7, P9, P13, P17, P18 (*n* = 5) *None: P2, P3, P8, P16 (n = 4)*

The number of each third-level category refers to the number given in the coding during the thematic analysis undertaken across all the phases of the reported experiences, and thus, corresponds to the ordinal numbering in the list of coding (see Supplementary Material).

The first one, “**1A-Bodily ownership**,” grouped mentions that alluded to the sense of having or owning a body, or the lack thereof. This sub-category included descriptions without clear mentions of a body or body parts, but also those that involved the awareness of owning a body without explicit bodily sensations. For instance, three participants described what we labeled as “*Weak embodiment*,” a sense of feeling embodied in their experience without strong bodily feelings (P13, P15, and P18). P16 described a “*Distorted*” bodily awareness, such as feeling their body as a cloud of energy. Moreover, this sub-category also included descriptions that lacked even this minimal sense of bodily ownership yet involved some sort of self-identification with an aspect of the experience. In these cases, while participants did not feel contained within some sort of bodily boundaries, or perceived their own body, there was a sense in which they felt their experience as their own, either while the experience was unfolding or afterwards. We classified these descriptions under the third-order category “*Minimal identification*.” Four participants described a sensation of bodiless awareness, involving feeling that they lacked a body (P7, P9, and P17), including a “sensation of nothingness” (P1). Other three participants self-identified themselves as “a sphere of light” (P2), a “speck of light” (P12), or “the void” (P8). Finally, P3 reported lacking a “sense of ego” during the experience—they described lacking any sense of themselves being within it. Nevertheless, they reported an awareness of a “tapping” or “pulsing” that occurred in this phase, and it was just afterward, during the report, that they assigned took this to be their own experience (see Table 2 for illustrative examples).

**Table 2:** Illustrative examples of the sub-category “***Minimal identification***” and the different ways in which this was instantiated.

1A-Bodily ownership	
***4-Minimal Identification***	
A sensation	*So, **this sensation of nothing** was letting me know that I was still in a dream, because I made the comparison to, I cannot feel any of my limbs. So, I know that I’m not just in bed right now with my eyes closed. **Because none of my body’s there**. So, **the sensation of nothing was actually letting me know that I was** still in the dream. (P1:26).*
Bodiless awareness	*And then, and then all of a sudden, there was just nothing I could not, **I’ve gone from, from my body**, I guess. And I’ve had other bodies before and this, this felt very, very, very different where I did not like there was no dream body no dream scene. No, no ANYTHING.* ***It’s*** ***almost like seems like a form about a body**. But it almost seems like you are, you are caught between, caught between somewhere where you are trying to get in and the physical, you are, you are somewhere else. […] And so, so I was able to feel that I guess. (P9:18).*
A sphere of light	*[…] **I no longer have an idea of a body a dream body** at that point (P2:37) And then **I [emphasis] became or was this just like this little ball of light**, […]. So like **I knew that the sphere of light was ME**, but also like the light that was around the sphere was me, […] (P2:36) Once I become the sphere, you are asking if I have any body perception? I do not have any at that point […] (P2:47) […] **having a dream body is just completely gone**. […] (P2:48).*

Note that for each report we have indicated the participant number and the description number in the report.

The second sub-category “**1B-Spatial self-location**,” referred to the sense of being somewhere within the experience, or to how one feels one’s location in the environment—the feeling of being located “somewhere.” In many cases (5/12), participants described having a feeling of being “in the nothingness” or being somewhere within the nothingness, yet with an “*Indeterminate*” location (P1, P9, P12, P15, and P18). We isolated the category “*Physical*” for those descriptions involving a sense of spatiotemporal location similar to that experienced during wakefulness. For instance, P7 described the experience of being in a room, which they took to be part of their dream experience and felt the distance between them and the walls. We also distinguished between “*Minimal*” spatial location and “*No clear boundaries*.” The former was constituted by descriptions involving merely the feeling of “being there” without references to a spatial location, including a location somewhere in the nothingness (P8, P13, and P17). The latter classified descriptions mentioning a self who has become part of the whole experience (P8, P12, and P16). Finally, two participants reported lacking any sense of being located within the experience whatsoever (P2 and P3; see Table 3 for illustrative examples of each sub-category).

**Table 3:** Sub-categories (second level) for the dimension “***1B-Spatial self-location***” and some quotes exemplifying each sub-category.

1B-Spatial self-location	
1- Physical	*I suddenly felt like **I was in this BUILDING**, like a factory (P7/1, 40). For once, I can FEEL [the space]. **I could feel the DISTANCE** from my awareness **in there, to the walls and the door, and so on**. And yes…**there are different points that make up this space**. […] (P7:47–48)*
3- Indeterminate	*So, **I’m still the same** as I was before. Except there is no relation to other things around me. So, I did not as the scenery disappeared. **I did not feel like I somehow move or anything**. Just, **I was in the same location**? (P15:51)*
4- Minimal	*It’s like, **there’s no beginning there’s no end**, there’s not like a locational type of thing (P17:22) **But I’m IN**, […] It’s because it’s just infinite (P17:23) **I…just I’m just there**. (P17:20)*
5- No clear boundaries	*It’s more like **I was the void**. […] (P8:44)* *It’s just total darkness. And you…, **there’s very little difference between you and what’s around you**. […] (P12:30)*
6- Absent	*But in that experience, **there was not even a sort of sense of me being a, a person or anything** like, you know, for…for me to say like, here’s an outside and here’s an inside, **it was just this sort of tapping** (P3:12)*

The third sub-category, “**1C-Perspective**,” intended to characterize the subjective point of view had during the experience, or the egocentric point of origin of the experience Some of the descriptions included in the sub-category “**1B-Self-location**” were also included here since those also mentioned the egocentric perspective of their experience. Two participants described a first-person perspective similar to that had during wakefulness, such as an “I” observing or perceiving the experience (P12 and P18), classified as “*Regular*.” Another two also had a similar sense of first-person perspective, yet their descriptions alluded to a “*Fluctuating*” point of view; they could “see” themselves from the outside (P18), or they could see in multiple directions (P2). However, the most prominent third-level category was that involving a “*Minimal*” sense of subjective perspective— a way in which the experience felt like happening from their point of view without this point or position being explicit. Two participants described it as being “inside” the experience, or as being “part of” the experience (P16 and P17). The other two described a non-ordinary visual experience, in which they could rather feel (instead of seeing) the presence of shapes or movements (P7 and P15), or as seeing blank (P8; see Table 4 for examples). Finally, as in “1B-Spatial self-location,” the experience of P3 did not involve any sense of subjective viewpoint and they did not recognize themselves as being part of it while the experience was taking place.

**Table 4:** Examples of quotes referring to a “*Minimal*” sort of first-person perspective of point of view and the different ways in which it is instantiated.

1C-Perspective	
***3- Minimal***	
Point of view as part of the experience	*(I: Is there a point of view that you are you present IN this light or are you looking AT that light….?) **No, I’m present IN that light** (P16: 9) Yeah, it was really like a bath in this energy and light. (P16:45) […] Yeah, having a BATH inside of this light (P16:48) […] **I’m INSIDE the experience** […] (P16:28, edited)* *Imagine like, **just BEING in the point of view of just like being in those colours** […] **But I’m IN, I’m in the point of view of like, anywhere I go**, […] I’m not like able to like TURN this environment or like OBSERVE it from like different points of view[..] I’m seeing and just like IN, **immersed IN these colours**. (P17: 23)*
Not ordinary “seeing”	*It’s like, if, in one, you are looking at a movie screen that has nothing on it. And in other, someone turns out, light […] Yeah*, ***it’s*** ***kind of like seeing white in front of me**. That’s not exactly what’s going on. But that’s the closest I can get to describing it (P8: 61–65)*

The final sub-category, “**1D-Agency and attitude**,” grouped descriptions referring to a sense of agency, the sense that one is the agent who generates or initiates action. We also included descriptions that were not so explicit about feeling oneself as being in control of the actions taking place in the experience, but merely as the subjective experience of having an intention. We isolated three different ways in which the sense of agency was instantiated. The first is an “*Active*” sense of self involving an agent taking control by either trying to keep their lucidity or awareness of their experience (P1 and P18), by actively engaging in exploring further their experience, or by manipulating their attention under their will (P7 and P13). The second was a “*Receptive*” agency involving some degree of lost control and an agent accepting this fact (P1, P7, and P15). Other participants said to have just adopted an attitude of not doing anything, of just observing or staying with the experience (P12 and P16). Finally, some of those participants also described how at times they felt to have “*Lost control*” and could not proceed with their intentions, either by not being able to go “deeper” to explore this state (P7), by feeling they could not move (P9 and P18), or by unintentionally transitioning to a different phase (P13 and P17). Note that in some cases these different sub-categories isolated can refer to the same experience and the same participant—an experience could have been described as having “*Lost control*” yet having a “*Receptive*” attitude toward it (see Table 5 for details).

**Table 5:** Quotes characterizing the dimension of “**1D-Agency and Attitude**” for three of the participants alluding to more than one third-level category in their descriptions (P1, P7, and P18) during the “nothingness phase.”

1D-Agency and Attitude	
*[…]. And that all went away and just disappeared. […] (P1, 22) And **I could always wake myself up at any point in time if I wanted to**. I could. **But I did not want to** (P1:25)* *And then I just remained there. And **I just tried to keep my awareness and my lucidity** with clear intent to not let it go…[…] (P1:10)*	P1 describes how the dream scenery disappeared, and that they knew they could wake up, but they did not, they accepted this state (**2- Receptive**). Then, they tried to actively keep their awareness during this state (**1- Active**).
*I had the intention before that…**I wanted to EXPLORE this state and to go as deep into it as I can**. And this still remained…(P7:73)* *[…] **I could not do that**; something was stopping me then. (P7-1, 52) […] **There was something holding me back**. And so, there was like an invisible barrier, but I **could not get through** […] (P7:76)* *Yes, at some point, **I noticed that I just cannot go there now. And so, I thought, Okay**. […] (P7: 89)*	P7 mentions how their intention was always to go “deeper” into the state and explore it (**1-Active**), yet when they tried, something was holding them back (**3-Lost control**), but they end up accepting it (**2-Receptive**).
*[…] the main thing that remains and that has been kind of prevailing since I became lucid is this **determination of maintaining awareness**. […] (P18:33) […] [The lucidity] remains and I think it’s because **I’m very determined to maintain lucidity** and not to, I do not want to like get IMMERSED in the dream, I want to stay aware […] (P18:60)* *And at the same time, it’s a bit strange, **because I cannot really like control it completely**. […]. (P18:50)*	P18 also mentioned having had the intention to maintain the awareness, and that this determination was kept thorough the experience (**1-Active**), however, in a sense, they could not completely control it (**3-Lost control**).

##### Sensations

There were different sorts of sensations reported during this episode of the experience. To explore more the differences between them, we grouped the sensations reported in three different groups: “**2A-Bodily sensations**,” “**2B-Kinaesthetic sensations**,” and “**2C-Non-modal sensations**.” The first two sub-categories are sensations that occurred within different sensorial modalities (including touch and proprioception). We distinguished between those sensations that explicitly mentioned contact within the body as “*Bodily sensations*” from those that might involve the body, yet not direct contact with it as “*Kinaesthetic sensations*.” Regarding the former, during the “nothingness phase,” we only found mentions of a lack of bodily feelings, mentioned by four participants (P1, P8, P9, and P12). It was only on other phases of the reports that we found mentions to “**2A-Bodily Sensations**,” including a feeling one’s body, or a body part, or feelings of touch. As for “**2B-Kinaesthetic sensations**,” most participants did not mention any in their descriptions during the “nothingness phase” and only some reported having a sense of their body position (P15 and P18), or a sense of being floating or suspended in the air (P1 and P18; see Table 6).

**Table 6:** Second- and third-level categories and the participants identified for each for the first-level category Sensations.

2. Sensations	
2A-Bodily sensations	***4- Absent*:** P1, P8, P9, P12 (*n* = 4)
2B-Kinaesthetic sensations	***1- Position:*** P15, P18 (*n* = 2) ***3- Floating/hanging or suspending in the air:*** P1, P18 (*n* = 2) ***4- Release tension*:** P17 (*n* = 1) ***5- A force/barrier*:** P7 (*n* = 1) ***6- Absent:*** P1 (*n* = 1) *None: P2, P3, P8, P12, P13, P16 (n = 6)*
2C-Non-modal sensations	***1- Modality-like:*** P3, P7, P13, P15, P16 (*n* = 5) ***2- As having material properties:*** P7, P15 (*n* = 2) ***3- As lacking anything*:** P1, P9, P17 (*n* = 3) *None: P1, P2, P8, P12 (in a different phase)*

The number for each third-level category corresponds to the number given in the coding for the thematic analysis (see Supplementary Material).

The most frequent sort of sensations described during the “nothingness phase” were those that participants said not to pertain to a sensorial modality or “**2C-Non-modal sensations**.” For instance, many participants (5/12) alluded to a feeling that could be said to be modal, yet it was not felt by any of their senses, and so we classified them as “*Modality-like*.” Some descriptions mentioned sensations that were tactile-like yet did not involve contact with their body (P3 and P16). Others mentioned an auditory-like sensation, such as a sound with no source (P13 and P15). One participant described them as vision-like sensations, different from ordinary seeing (P7). We also included in these sub-category descriptions of sensations that were difficult to categorize and that, in some cases, had an esoteric-like tone. These include descriptions by P7 and P15 about “the nothingness” as if having some physical or material properties, such as “the nothingness” or “darkness” “leaking from the door” (P7) or the “tiny movements” forming the nothingness (P15). In contrast, P1, P9, and P17 described this state as merely “feeling nothing” (see Table 7 for illustrative examples of “**2C-Non-modal sensations**”).

**Table 7:** Illustrative quotes referring to the different third-level categories for the sub-category “2C-Non-modal sensations.”

2C. Non modal sensations	
**1- Modality-like**	
**Tactile-like:** While P3 described what could be taken as a tactile or bodily sensation in their chest, in different parts of their report they stressed how not only do they lack any bodily sense during the experience, but also a sense of “a self,” so all there was it was just this tapping or pulsing.	*I’m feeling this sort of sensation in the chest with no sense of, of MYSELF feeling (P3:4) […] it was just a sort of bare Morse code, code like pulsing at the chest. […] (P3:9) **It wasn’t like, as if someone were tapping the…my chest**, you know what I mean? It wasn’t at the surface of my chest. It was INSIDE the chest. (P3:11) So if I were to translate it in terms of like, audio information, **it’d be something like ta-ta-ta-ta-ta, ta-ta-ta-ta-ta-ta, ta-ta-ta-ta-ta-ta-ta, ta-ta-ta-ta-ta-ta-ta-ta…** Now, of course, **I’m not HEARING anything**. […]. It was just this sort of like, a tactile sense in that rhythm. (P3:10)*
**Sound-like:** Both P13 and P15 described a sound that overtook the experience. P13 described the sound as being part of the experience, as a sound, they could feel, yet not hear as such. P15 described that after the dream scenery disappear, they were some “tiny motor movements,” which they describe by referring to the sort of buzzing or noise that the TV does when the signal is out.	*[…] the sound like had- **like an EMBODIED experience of it…**(P13:34) I could, **I guess like FEEL the sound** if that makes sense. […] (P13:35) […] And the sound was the overwhelming part of it. And it was like an all-encompassing sound. So not like a dream of like a bird chirping, but just like **the STATE IS THE SOUND**. (P13: 9)* ***Like the television screen!** You know when it goes bad. It’s similar to that. But smaller and less irritating than the TV screen. You know, they are and they could have **the “shshsh,” but a lot quieter** (P15:49)*
***2- As having material properties***	
Something is there	*[…] I could already feel it before that **there is very thick darkness inside that room behind the door**, **it kinda came leaking out of the door** (P7: 42) It was like **I was feeling it through the door**. (P7:55) […] But, am…That there, it’s not only DARKNESS, it’s that there ISN’T anything, it’s NOTHINGNESS. And so, I felt this kind of VOID in there. (P7:57) [the nothingness] **felt very ABSOLUTE…and…somewhat EXPANDING** […] (P7: 59)* *it feels very, **very MIGHTY**. […] (P7:70)*
A sense that can be seen; it could also be coded as a visual-like modality	*(I: And so is there a feeling that you are that there is a space that is dark? And **does not have anything visual** in it? Or is there a feeling that you are in nothingness?) P: Hm!! I think definitely did not have the feeling of emptiness. **I still had a feeling of something being there**. Me being there. **And also the tiny movements…existing**, you know, it was the fact that I perceived them was the opposite of they are just being nothing. **No, it was something**. […]. **I think that tiny movements, was there something as opposed to nothing** (P15:47)* *Yes. I see the tiny movements. So, like, the tiny movements are, in a way, the fact of fading and disappearing. But **at the same time, it’s something that I somehow see, I, or maybe, let us say, perceive**, because as I see it, it does not really have like a shape or a colour. But it’s still something that moves, something that is happening (P15:33, earlier quote from a different phase)*
**3- As lacking anything**	
*But then **once [the senses] disappeared, I had nothing**. And I was just floating in nothingness (P1:3) And that just feels like total nothingness. Like just emptiness. (P1:33) Because **the only sensation that you have, if you could even call this sensation would be this sensation of nothing** (P1:26)*	

##### Visual Experience

Another prominent feature of the “nothingness phase” was the absence of any visual imagery. For all participants, the transition into this episode ended with the absence of complex visual imagery (understood as visual perception or imagery like ordinary visual experience during wakefulness). We explored this in more detail and classified the descriptions of their visual experience as “*Loss of imagery*” and “*Absence*.”

The sub-category “*Loss of Imagery*” referred to descriptions from participants mentioning that, in a way, they were able to perceive, yet there was not anything to be seen. P1 described this as “blackness” and P18 as lacking color or light, while P12 described it as “darker than being in a dark room.” P8 described the absence of anything “as white,” since there was nothing there, including black. The other three participants seemed to describe this state as involving some sort of perception of light, flashes, or colors (P2, P16, P15, and P17), yet it was difficult to gather from their descriptions to what extent this was experienced as an ordinary visual experience. For instance, P2 mentioned to “look” at a “sphere of light,” which they self-identified with. P15 described it as the experience of being with the eyes closed and perceiving some “little flashes” or “holes.” P16 also described the presence of a light, which changed color, but like P2, they took themselves to be “in” this light. Finally, while P17 mentioned “seeing” “spirals, colors and shapes,” they also mentioned they were in “the void,” a state of “blackness” in which they could not see anything.

The sub-category “*Absence*” includes three other descriptions emphasizing not only the lack of imagery but the lack of any sense of vision. For instance, P3 contrasted this state of nothingness to another experience they described as a “dream lacking visuals,” a dream in which they were not seeing anything. P7, P9, as well as P17 who mentioned the presence of “colors and shapes” described this experience as not ordinary “seeing.” Finally, P13 did not allude to any visual experience during this state (see Table 8 for illustrative quotes).

**Table 8:** Illustrative descriptions for the category “***Visual experience***” and the sub-categories isolated during the “nothingness phase.”

3. Visual experience	
**(3) Loss of imagery** (*n* = 8)	
Absence of imagery; blackness	***Everything is…is black** (P1:11)* ***[…]** like, actually **being in a dark room in which there is…absence of light** (P12:18)* ***It’s*** ***just kind of colourless and lightless** (P18:3)*
Absence of imagery; whiteness	***There’s*** ***just white**, […] **For lack of anything else, I mean, it’s not black**. (P8:61–65)*
Absence of imagery: light	*[…]* ***it’s*** ***almost like I was LOOKING at the sphere of light too** (P2:45)* ***[The light]** has no source*, ***it’s*** ***everywhere** (P16: 8)*
Absence of imagery; flashes/colors	***[…] it looks like white against a colour. So, I guess they are like a little, they could be holes. But I rather describe them as little flashes**. (P15:49)* *[…] almost as like this water’s being shaken up. **And like the colours of the ink are just being like, shifted around and going around each other and moving all which way**. […] (P17:23)*
**(4) Absence**: P3, P7, P9, P17	
***And I could tell the difference between, like, you know, having my eyes closed, and just not being aware of any kind of visual information** […] There was NO sense of vision (P3:8)* ***Not like seeing with my EYES** (P7:43)* ***And, and you had, you had no sensory perceptions**, and you had NOTHING. (P9:15)* ***just SEEING is not, is not a thing there** (P17:28)*	

##### Emotion

The category “**Emotion**” aimed at isolating descriptions that contained mentions to the “*Presence*” or “*Absence*” of an emotional tone of the experience. Originally, this category was further broken down into “affective valence”—the subjective attribution to the experience or different aspects of the experience made by the participants—but eliminated afterward given its very few mentions. Six participants described the state of nothingness as accompanied by good sensations such as “feeling good” or “refreshed” (P9), “happy” (P16 and P17), “glad” (P18), or “relaxed” (P12). The other two described the state as “contentment” (P1) or “acceptance” (P15), which was accompanied by a “slight sense” of “disappointment” for having lost the dream scenery. The other three mentioned the absence of any feelings or emotions. P2 reported not remembering having had any feelings, whereas P8 described how a relaxing and “narcotic feeling” they previously had when transitioned into the void was lost. P3 described their feelings as “flat,” as lacking emotion. Finally, P13 did not allude to any emotional sensations, either their presence or absence.

##### Attention

The category “**Attention**” grouped the different ways participants were aware of any object or content of awareness, that is, the sort of attention had toward their conscious experience. We distinguished amongst: “*Focused*,” “*Dynamic*,” “*Resting/Vague*,” and “*Wide, unfocused*.” As in the case of “Agency,” these sub-categories are not mutually exclusive, and oftentimes the descriptions could be characterized as involving more than one. Except for P1, all other participants described the type of attention had during the “nothingness phase.” The most common type, mentioned by six participants, was what we classified as “*Wide, unfocused*” attention. This subcategory illustrates how the “nothingness phase” merely involved an awareness, yet “nothing to be aware of” (P8). This was described by some participants as the absence of thoughts or feelings (P2) or as nothing to pay attention to (P13 and P17), a state of “just being conscious” (P3) or “feeling ultra-aware” (P9). This wide attention was slightly different from what we characterized as “*Resting/vague*,” an attention still involving a distinct object of attention—such as attending to a specific feature of the experience—yet experienced as “implicit” by the participants. For instance, P15 said knowing the “tiny movements” were still there even if they were not paying “explicit attention” to them. This type of attention was also distinctive from that characterized as “*Focused*” which alluded to the distinct awareness of one’s own thoughts or feelings, described as “self-reflective” by P13 and P15. Finally, P7 described how they were aware of having changed their focus of attention and how this fluctuated thorough the experience (see Table 9 for details).

**Table 9:** Illustrative examples of each of the types of “**Attention**” isolated from the phenomenological analysis.

4. Attention	
(1) Focused	*Like, there’s more, I guess, like, like, **self-reflective thinking there**. Not as much about like, like me as a person, **but just as thinking that’s happening**. (P13:39)*
(1) Focused; (3) Resting/vague	*So, **I…had these thoughts. They were a realization that the scenery was disappearing**. They were a direct response to what I was saying. They were in Slovenian. “**Quickly try to remember something else. Where can you go?**” (P15:56–57)* ***I do not remember right now, particularly focusing on the tiny movements as such in that moment. I know there were there. They were constantly part of the experience. But I wasn’t explicitly focusing on them**. I was somehow letting myself…**Ha! Observing. So, observing**. But observing is not like, just looking and focusing on them. **Observing is looking at wanting to give something to it, to give it meaning, to figure out what it is**. Just looking would be just letting into the visual perception, I guess. (P15:80)*
(2) Dynamic	*I felt that, that **my awareness is everywhere inside this room, but it’s not always in all places, but it changes**. (Mhm.) Like it’s here and there and moves around. (P7:36) […] [the transition] is like going into different directions of, am…of possibilities that are in my awareness somewhere. The experience of this factory setting was the main place of my experience, then…there would come up some thoughts while being there. And if I just…**if I then went with these thoughts, I would do somewhere else**. (P7:51) **when the door opened, I was JUST with my awareness somewhere else**. (P7:53)*
(4) Wide, unfocused	*Because **you kind of go from awake to all of a sudden…AWARE** (P9:30–31) Am…In that state, it’s just…just like, yeah, **just pure consciousness** where you are there and at first, I just relaxed in…and just relaxed in it (P9:12)* *No*, ***there’s*** ***nothing…there would be like really NOTHING to pay attention** to, I guess. **Unless you can count, like being attentive to nothing** (P13-2:30)*

##### Awareness of the State

Finally, the category “**Awareness of the state**” aimed at capturing what the participants took the overall experience to be. We classified mentions of knowing that one was: (1) sleeping or in bed, (2) dreaming, and (3) in a state of awareness. For most participants (8/12), there was an awareness of being aware, or an awareness of their awareness, a sense in which they knew that they were conscious (P2, P3, P7, P8, P12, P13, and P17). In some cases, the descriptions mentioning the awareness of the state also alluded to their experience of a “self,” such as a sense in which they knew they were in the experience, or that they were in there (see “Sense of Self”). Similarly, other descriptions on this sub-category were also coded as “*Wide, unfocused*” in the category of “Attention,” described as the sort of awareness involving attention that does not have a distinctive object of awareness. Other participants explicitly mentioned how they took this “nothingness phase” to be a dream experience, and so, they knew they were dreaming (P1, P18, and P15). Finally, the other two (P7 and P13) said they knew they were in bed or sleeping, yet this knowledge was in the background, they did not “think about it.”

*“Okay, so it's not that you consciously knew ‘Oh, I am asleep’. It's more that I need to do this otherwise I would wake up.”* (P13:28, emphasis added)

##### Overall Synchronic Categories

The phenomenological analysis for the “nothingness phase” resulted in the isolation of 6 first-level or higher-order categories. From those, the most representative third- and second-level categories, which were present in more than half of the participants, were “*Minimal identification*,” “*Loss of imagery*,” “*Presence of emotions*,” and “*Knowing they are aware*” (see Table 10 for a detailed summary).

**Table 10:** Summary of mentions provided to the most frequent third-level and second-level categories.

First-level category	Second-level category	Third-level category	Mentions/total	No mentions/total
1. Sense of self	Bodily ownership	Minimal identification	8/12	0
	Spatial self-location	Indeterminate	5/12	0
	Perspective	Minimal	5/12	2/12
	Agency	Lost control	5/12	4/12
2. Sensations	Non-modal sensations	Modality-like	5/12	3/12
3. Visual experience	Loss of imagery	-	8/12	1/12
4. Emotions	Presence emotions	-	7/12	2/12
5. Attention	Wide attention, no focus	-	6/12	0
6. Awareness of the state	Knowing they are aware	-	8/12	0

### Explorative Quantitative Analyses

#### Individual Self-Ratings

The calculation of means for the self-ratings carried out by the participants about the degree of vividness, completeness, articulation, and accuracy of the recollection of the experiences reported in the interview (during the spelling exercise and the potential experience of objectless dreamless sleep) revealed similar ratings between the different dimensions of the recollection for the experiences in the first part and second part of the interview session (see Supplementary Material). The overall mean for “vividness” and “recollection” was quite high, while the overall mean for “articulation” was slightly lower than the rest. For “invention” the overall means were quite low, but for this dimension, the score was inversed (0 = no invention; 10 = a lot if invention).

#### Intercoder Agreement

Fleiss’ Kappa was run to determine the degree of agreement between the three coders (the two external researchers, and the main author) on their classification of the different categories isolated in the thematic analysis. By accounting for those descriptions that alluded to more than one code (or a subset of category and subcategory), a total of 220 classifications were considered for the statistical analysis. Fleiss’ Kappa (*K* = 0.481) indicated a moderate level of agreement amongst the three coders (cf. Fleiss et al., 2003). We performed further analysis to investigate the level of agreement between different coders, finding a good coefficient between Coder 1 (external researcher) and the main researcher (*K* = 0.627), and lower but still moderate between Coder 2 (external researcher) and the main researcher (*K* = 0.458). The analysis showed a fair agreement between both external researchers (*K* = 0.357; see Supplementary Material).

Finally, another Fleiss’ Kappa analysis revealed the categories and subcategories with a higher intercoder agreement across coders for the “nothingness phase.” “*Emotion: Absence*” had a very good Kappa coefficient (over 0.8). We also found a good coefficient (between 0.6–0.79) for: “*Agency; Active*,” “*Agency; Receptive*,” “*Bodily sensations; Absence*,” “*Non-modal sensations: Modality-like*,” and “Awareness of the state; *Knowing they are sleeping and that they are in bed*.”

## Discussion

This second phase of the research study “*Objectless sleep experiences*” offers one of the most detailed and extensive phenomenological characterizations to date of conscious experiences during sleep described as lacking a distinct object of awareness. From the phenomenological interviews conducted, we selected the reports by 12 participants describing what they took to be an experience of “nothingness” while sleeping. This episode followed either the awareness of the disappearance of a dream, the ending of their sleep-mentation or was experienced suddenly after falling asleep without previous recollection of events (see section Diachronic Structure). Our analysis yielded the emergence of six experiential categories with their corresponding second- and third-level categories which shed light on the phenomenological blueprints of such a state. The present results add up to previous research investigating the phenomenology of such an experience (see Alcaraz-Sanchez, 2021). In this last section, we discuss the main findings by relating them to previous empirical and theoretical research in the area, highlight the shortcomings of the study and introduce some pointers as to where to proceed with future research.

### Alterations on Self-Awareness

One of the most prominent features of the state of nothingness described by the participants was the disruption of their self-awareness, understood here as one’s self-perception within the experience. In the literature, the phenomenology of sensations referring to how conscious experience feels subjectively as one’s own is widely known under the term of “sense of self.” However, this notion is also heterogeneously defined in the literature, ranging from the subjective feeling of “I” or “mineness” (see Zahavi, 2005), the feeling of “being someone” (Metzinger, 2003; Blanke and Metzinger, 2008), or the feeling of being the subject of the experience (see Gallagher, 2000), to mention some. Similarly, the descriptions made by our participants alluded to different dimensions of this “sense of self” distinguished in the literature (see Millière, 2020 for a review), which were recognized in the clustering process during our phenomenological analysis. The analysis revealed that, while all participants described a self-awareness different to that had during ordinary wakefulness, involving in most cases an experience lacking any bodily sensations or bodily experience, with the exception of two, most of them experienced themselves within the experience—there was a way in which they felt to be in the experience, even if they said to lack the experience of a body or bodily sensations. Here, we explore the most frequent sub-categories for self-awareness or sense of self: “Minimal identification” to describe “Bodily ownership.”

Following some accounts in the literature, the sense of bodily ownership can include an experience without explicit mentions to the body, or body parts (see De Vignemont, 2013). Similarly, some other accounts also understand that one could feel their experience as their own, even if their experience does not involve an explicit sense of being contained within certain bodily boundaries or perceive one’s body as one’s own (see Gallagher, 2017). Given this understanding of bodily ownership and its relationship with the sense of feeling oneself as the subject of the experience, we might want to consider the sub-category “Minimal identification” as involving an experience in which one feels oneself as the subject of the experience or had a minimal sense of being in the experience. Such an account could explain the presence of a minimal sense of self as the experience of being someone, or subjectivity, even in those reports that described a lack of an explicit bodily sense or were said to be “bodiless.” In the literature, the existence of these so-called *bodiless* states, or states that do not involve the phenomenology of bodily ownership, suggests that self-awareness does not always necessitate the experience of oneself within a body (see Millière, 2020). For instance, research on self-awareness during “*bodiless dreams*” (see LaBerge and DeGracia, 2000; Cicogna and Bosinelli, 2001; Occhionero et al., 2005), dreams in which the dreamer says to exist as a “disembodied entity,” suggests the existence of a minimal sense of self in the absence of bodily awareness:

*“I was inside a gigantic photocopying machine. I knew I was inside, as an abstract entity, as a mind, I was the machine, so I couldn’t see myself.”* (Cicogna and Bosinelli, 2001, p. 32, emphasis added)

In this brief report, the dreamer says not perceiving themselves in a regular way; nevertheless, they are able to feel they are in the experience, instantiated by a sense of self-location (“I was inside…”), but also, to self-identify with something (“as an abstract entity,” “I was the machine”). Similarly, participants like P2 said to “no longer [had] an idea of a dream body,” yet still had a sense of minimal self-identification within the experience, in this case, having been a “sphere of light”:

*“…when I was the sphere of light, it was that there was no sense of…of self, like there was just, it was just the sphere of light. So, it was, it was almost like, how do I explain that, but…it was maybe, a different…a different me, just not the me that I think about when I'm awake.”* (P2:41; emphasis added)

Such reports seem to talk in favor of authors understanding the experience of bodily ownership as not amounting to bodily sensations (see De Vignemont, 2013), however, we should consider further whether reports of this kind do in fact involve a minimal sense of bodily ownership given by this minimal self-identification with something (i.e., being the “machine,” or “a sphere of light”). Similar reports are found in individuals experiencing “asomatic” out-of-body experiences (Metzinger, 2013, p. 4) which are described as disembodied experiences in which one feels as being a “ball of light,” or a “point in space” (see Alvarado, 2000, p. 186), but also a “gaseous ball” (see Rabeyron and Caussie, 2016) or as their body having been “melted” (see LaBerge, 1985). Other descriptions provided seemed to account for certain bodily ownership given by other elements of the experience, such as the sensations had. Some descriptions were more explicit than others, like P13 mentioning a bodily feeling given by an “inner sound.”

*“[…] And it was like an all-encompassing sound. So, not like a dream of like a bird chirping, but just like the STATE IS THE SOUND (P13:9) Yeah because the sound like had like an EMBODIED experience of it…(P13:34) I could, I guess like FEEL the sound if that makes sense. […] That was part of the experience of the sound. So, I think before the sound, I wasn't thinking about the bodily sensations.”* (P13:11, emphasis added)

Finally, there is the case of P3, who reported a lack of sense of “ego” during this episode and described a sense of “tapping” or “pulsing” as the only thing present during the experience.

*“There was not even a sort of sense of me being a, a person or anything like, you know, for…for me to say like, here's an outside and here's an inside, it was just this sort of tapping.”* (P3:12, emphasis added)

Nevertheless, while P3 were not aware of themselves being aware of the tapping (i.e., an awareness of the tapping as something that was happening to them), they remember having been aware of the tapping happening:

*“But I was definitely CONSCIOUS. I was definitely, there was some consciousness there, there was some sense that this tapping was happening. It was just that, that sense was very bare bones.”* (P3:26, emphasis added)

One might wonder to what extent this was a truly selfless experience—an experience that lacked a sense of self whatsoever—or there was still a minimal sense of self involved. For P3, it was just after the experience ended that the “tapping” was assigned as something that occurred to them, yet while the experience was unfolding there was an awareness of such tapping happening. Research on bodiless experiences during self-boundary dissolution in meditation could shed light on this sort of experiences in which one lacks a sense of ego, yet one is conscious of their experience unfolding. For instance, Ataria (2015) presents the following example of a meditator engaging in formal practice describing how they shift from being aware of the sound of an ice-cream truck entering their ear to becoming aware of just the sound:

*“And then I observed that the object itself, the fact that it was an ice-cream truck, disappeared. The next thing was the location. First the object itself disappeared, the so-called ice-cream truck. Then the location, in other words, distance, disappeared, and I began to focus on the sounds that entered my ear. And there was a sense that it was no longer in the ear, but it was in the mind, that it was …the hearing consciousness…arising in the mind. At that point there was no location; I would say that the location was inside of me, and there was no object. There was a very small object inside my mind…It was pure sound—pure sound that was not associated in any way to a thing. (M. K.).”* (Ataria, 2015, p. 1134)

From reports like the previous, Ataria has suggested that the sense of bodily ownership can be given by the sensations experienced, such as the sound reported in the previous report—the sensation defines the boundary between myself and the rest (Ataria, 2015, p. 1133). Similarly, other researchers have suggested that the sense of bodily ownership is not confined within one’s body and that a minimal sense of self can remain in absence of bodily ownership (see Ataria et al., 2015; Nave et al., 2021).

### Lack of Sensory Perception

Another striking feature of the “nothingness phase” was the absence of any visual perception. However, from some of the descriptions provided, we might wonder whether there was in fact a total absence of visual experience. On one side, some participants described this state as one that lacked a sense of vision altogether. P3 compared this state of nothingness to a dream in which they could not see anything, mentioning how different it felt seeing an absence of vision from lacking vision at all. On the contrary, some other participants did provide descriptions of what seemed to involve some visual perception. For instance, P15 compared it to having one’s eyes closed and said to perceive “tiny movements” or “little flashes,” which at times were described as involving visual experience. Similarly, others described the perception of “light” or a “source of light” (P2 and P16).

From those different reports describing the absence of visual experience, we can make different speculations. One is to suggest that at least some participants were indeed having some visual experience, and thus, what they were perceiving was the lack of any visual percepts. In a way, there was an experience of “absence,” such as when one is in a completely dark room, or with the eyes closed. Results from sensory and perceptual deprivation research can offer some insights into the phenomenology of perceiving lack of visual stimuli. Several studies show how prolonged periods of sensorial deprivation can give place to simple hallucinations, such as dots, patterns, and lights (see Merabet et al., 2004; Lloyd et al., 2012) as well as more complex hallucinations (see Heron et al., 1956; Zubek et al., 1961; Heron, 1965). Similar reports are made during states that only lack visual experience, but not perception altogether. For instance, some meditators provide reports of the so-called meditation-induced light experiences (see Lindahl et al., 2014, 2017), the perception of lights while engaging in meditation with the eyes closed. From these findings, we could take the perception of simple visual precepts such as the “tiny movements” described by P15 or the more overwhelming “bath of light” by P16 as instances of hallucinatory experiences.

Another alternate reading that could be made, is that the objects perceived (including lights, or flashes) are indeed veridical percepts. For instance, Mavromatis (1987) has suggested that experiences of lights or patterns had in environments with sensorial deprivation could be taken to be of retinal nature. Similarly, we could speculate that some descriptions allude to the perception of external stimuli. It is important to note that since our study was not carried out in an experimental environment, we are not able to determine in which sleep stage participants were (or whether they were sleeping at all). Moreover, even while sleeping we are not totally occluded from processing external stimuli and some sleep stages allow more perceptual processing than others. There is also research indicating a connection between the presence of altered states of consciousness during sleep, such as pre-lucid dreams (Tyson et al., 1984), sleep onset hallucinations (Takeuchi et al., 1994), sleep paralysis and false awakenings (Mainieri et al., 2020), and higher levels of alpha activity, which in turn has been linked to more external sensory perception (Tyson et al., 1984; Conduit et al., 1997; Darracq et al., 2018). Thus, we could speculate that during the state described by our participants, there was some integration of external stimuli which could have given place to some of the characteristic “Non-modal sensations” reported, such as those involving what could be considered as esoteric or mystical-like elements like “feeling the nothingness” or a “felt inner sound” (elements that could be taken to go above or beyond the realm of “reality” or what is possible in the natural world). Further comparison between the phenomenology of sensations had during episodes of objectless awareness during sleep and other altered states of consciousness paired with their electrophysiology could help us to understand better the role that processing of external stimulation might be playing in those states.

Finally, regarding the relationship between the lack of sensory perception and those descriptions grouped under sub-category of “non-modal,” we could also interpret those descriptions as alluding to certain sensations that cannot be accounted as pertaining to a particular sensorial modality. In the micro-phenomenological literature, sensations that are not about a sensorial modality have been regarded as “transmodal feelings” and characterized as “fuzzy feelings which do not fall within a particular sensorial modality” but that have properties that are “transposable from one sense to another” (Petitmengin and Lachaux, 2013, p. 3), such as “temperature, texture, intensity, rhythm and movement” (Petitmengin, 2007). Thus, we could speculate that a subset of those descriptions talking about “tiny movements” or “feeling the nothingness,” refer to the way in which this phase of “nothingness” was perceived: these elements in those descriptions regarded as “non-modal” are merely placeholders for describing one’s experience.

### Awareness of One’s Awareness

Lastly, we shall consider the feature of the state of nothingness as a state in which one is aware in absence of an object of awareness. This description itself seems at first contradictory since *awareness* is usually taken to be *of* or *about* something and does not seem intuitive to say that one can be aware without anything to be aware of. When asking participants to report a sleep experience they took be objectless, they described a state in which there was either nothing to be seen (visually), felt (bodily perception), or no mental activity (thoughts). As P8 describes:

*“I am aware not in the traditional sense. Traditional sense would have to involve some thing you can relate to, right? Time or thought. You know, you're definitely crossing into esoteric thought here where…there is…you're there, there's an awareness. But there's nothing to be aware of….”* (P8:65, emphasis added)

In the Dzogchen tradition in Indo-Tibetan Buddhism, such states of awareness are understood as states of non-dual awareness or non-duality; a state of awareness that lacks the subject/object structure of ordinary wakefulness (see Dunne, 2011, p. 262). Nevertheless, states of non-dual awareness are taken to be states of consciousness, states in which we are merely aware in virtue of their reflexivity; they have the property of “referring to themselves” (Williams, 2000). For some authors, states of non-dual awareness are states that do not necessitate second-order representations to be conscious (either a representation of itself or something else as an object; see Josipovic, 2019). As such, states of non-dual awareness are understood as states of intransitive consciousness, states in which one is conscious in virtue of having access to the phenomenal character of their experience.^5^

Nevertheless, upon further exploration of the interviews, we can identify some “objects” of awareness even if the descriptions provided seem to involve a different sort of awareness to that had during ordinary wakefulness. For instance, as we showed in the previous subsections, there was a way in which one was aware of lacking vision during the “nothingness phase” (there was an awareness of the absence of imagery), or that one was aware of the absence of bodily sensations (which in some cases, were accompanied with different sort of non-modality-based sensations). Similarly, some participants also described the presence of some thinking, yet they did not take this to be an instance of an awareness of one’s thoughts. This could be because they were aware of the process of thinking itself (the fact that they were in a state of thinking), yet they were not aware of the exact *content* of those thoughts. What those descriptions aimed at describing is how it felt to be aware of one’s own awareness, and whether that included an object of awareness other than their own awareness. In the micro-phenomenological literature, the notion “attentional disposition” is used to describe this process of self-reflection about one’s subjective experience—the way in which one becomes aware of one’s own experience (Petitmengin and Bitbol, 2009).

Some other participants, in particular, those classified in Diachronic Structure 1 (see section Phenomenological Analysis), took a slightly different meaning of what “being aware” meant. In this case, they related it to the experience of “lucidity,” similar to that had during lucid dreaming—they were aware of the fact they were in a dream, yet one that lacks dream scenery:

*“Because there it was the only thing that was left, like in the scenery disappeared, but my lucidity, the knowledge of being in a dream, didn't. (P15:69,70). I connect this to the fact that I knew that I was dreaming. So, in this mode disappeared in the moment we described earlier with the doubt, but then it was there all along. So, this is I could say instead of this mode, just being lucid.”* (P15:68)

Further theoretical work should investigate whether we should distinguish between an awareness of one’s own awareness, the awareness that one is dreaming, and the awareness of a dream that lack visual experience, or whether all those experiences should be accounted under the term “lucidity.” A good starting point would be relating phenomenological descriptions from this “nothingness phase” to those found during “open-monitoring” meditative practices, a style of meditation aiming at dropping attention toward any object of awareness and, instead, sustaining attention to the experience itself (Lutz et al., 2008, 2012; Dunne, 2015) Examples of meditation in this style are the Shamata and Mahamudra meditation (see Dunne, 2011) which are regarded as “objectless meditations” by some authors (Lutz et al., 2012), as well as Samadhi, which has also been related to the attainment of “objectless” states (see Millière et al., 2018 for a discussion). Moreover, some empirical research on case studies of expert meditators showing a state of “content-free” awareness indicate a similar experience of awareness to that of our participants (Winter et al., 2020). Other recent empirical research has also investigated systematically the relationship between experience with different meditation practices and the experience of “pure awareness” or “minimal phenomenal experience” and the different features characterizing such a state (see Metzinger 2020; Gamma and Metzinger, 2021).

### Strengths, Shortcomings, and Future Directions

The phenomenological interviews conducted in this study yielded extensive and fine-grained reports of experiences that were taken as involving the awareness of “nothing” or an awareness lacking a distinctive object of awareness by the participants. The interviews facilitated the exploration of dimensions and aspects of the experience that were previously unnoticed by the participants but, also, facilitated the gathering of descriptions that could have been difficult to obtain otherwise without guidance, given its ineffable character. Previous research has stressed the importance of how asking participants about specific aspects of their dream might impact the reports gathered (Nielsen, 2010). For instance, some studies have shown an increase of reportable emotional content during dreams after changing the scales used to self-assess emotions (Merrit et al., 1994). Similarly, other researchers have claimed how the lack of reports on experiences with minimal content during sleep might be hampered by the sort of questions prompted to participants when awakening (see Thompson, 2015). There is also the issue on how training in particular methods or experience practicing introspection might affect the quality of reports. Several studies provide evidence on how meditation practitioners, which usually have more experience in attending the qualitative character of their inner experience, provide more accurate objective introspective reports than no meditators (see Sze et al., 2010; Fox et al., 2012). Other authors have also suggested training participants in different techniques to increase the granularity of reports (see Windt, 2013 and Solomonova et al., 2014 for a discussion). This practice of collaborating with research participants to obtain better subjective reports has been used in the “neurophenomenological” framework to facilitate the comparison between first-person and third-person data (see Lutz and Thompson, 2003). The micro-phenomenological approach takes this principle of training participants into the method to facilitate the exploration of aspects of the experience that otherwise would have been unnoticed (see Petitmengin, 2006). In the present study, the micro-phenomenology inspired interview protocol helped participants to further their recollection by focusing on a particular experience had in a specific space and time, moving them away from generalizations and judgments about it. Thus, we would expect that further systematic studies using micro-phenomenological tools to result in the gathering of additional first-person reports on objectless experiences, either during sleep or during other conscious states.

Nevertheless, it should be noted that, in most cases, the interview process did not lead to a total evocated state, as detailed under the MPI guidance (cf. Petitmengin, 2006). There is a question as to whether this poorer evocation was due to the nature of the experience we are targeting, an experience occurring during sleep which is meant to be about “nothing,” or because there was a significant lag between the original experience and the interview session. Further experimental research on this phenomenon should be conducted in a sleep lab where not only participants can be interviewed just after awakening, but an adequate assessment of their sleep can be made. Moreover, conducting this sort of research in an experimental setting will help by shortening the temporal lag between experience and report (see De Vignemont, 2013) and thus, to meet with the gold standard of dream research of facilitating reports that are as close to the experience as possible. Although this might prove challenging, given the rarity and spontaneity of these sorts of experiences, future studies should investigate whether possible forms of objectless awareness like the one described here can be trained or be induced, like lucid dreaming (Voss et al., 2014; Sparrow et al., 2018; Aspy, 2020; Blanchette-Carrière et al., 2020).

The interview protocol took onboard one of the main tenets of the MPI method, which is to conduct “content-free” questions—questions that do not aim to influence the interviewee’s answers. It is standard practice with the MPI method to not ask triggering questions about elements that the researcher wants to investigate (unless the participant has previously mentioned those). However, as with any other qualitative research tool, the influence of the researcher’s previous conceptions or judgments cannot be totally avoided. Thus, not only the researcher can influence the answers by asking leading questions, but it can also occur more implicitly by participants themselves assuming that certain responses are expected or encouraged. For instance, one of our participants described how during their experience in “the void” they realized this was the sort of experience the main investigator was after. Similarly, those participants having previously experienced the targeted phenomenon might have chosen to talk about a distinctive instance of such phenomenon by focusing on the most striking or dramatic features. In psychological research, there is a debate as to what extent this behavior change is indeed a phenomenon and whether it impacts the research results (for a discussion see McCambridge et al., 2012). Participants are also influenced by their previous preconceptions and beliefs which might affect the answers provided. Most participants were acquainted with the sort of experience we were investigating, either because they had experienced it before or had learned about it. As we presented in the introduction, this sort of awareness in absence of any distinct object of awareness during sleep is considered by several contemplative traditions as one of the ultimate states one can reach in meditation, and thus, is taken by many as proof that one is a very skilled practitioner. Such considerations can be found under practices such as Yoga Nidra or luminosity yoga which guide practitioners to reach a state of “clear light,” a state usually considered in Indo-Tibetan teachings as one of the highest states of awareness (Mason et al., 1997; Travis, 2014). As such, participants embedded in this sort of practices might have been influenced by their expectations about the experience, and thus, we might question whether their reports are about what they felt during the state or what they took the state to be (see the issue of “Embodied theory contamination” in Metzinger, 2019). While in qualitative research we cannot completely remove participants’ preconceptions and judgements about the experience reported, we attempted to overcome this challenge by exploring in-depth their phenomenology, thus guiding participants out from descriptions that might contain judgements and evaluations of their experience and inviting them to explore the subjective character of the experience. Similarly, we should also acknowledge the difficulties and challenges by the participants to properly articulate their reports. Given that the targeted experience was frequently described as ineffable, most participants found it difficult to describe its elements. While the self-ratings provided by the participants about the recollection process were merely intended to be illustrative, were also useful to encourage participants to reflect to what extent they may have invented some of the elements on their reports (i.e., they were fabricated during the recollection, but were not part of the original experience), or how complete was their recollection (i.e., some aspects of the original experience were missing). The lower overall means in the dimension of “articulation” was due to lower scores provided by two participants who explained the difficulty of providing experiential reports on their second language, and thus, not necessarily meant that all participants found it difficult to describe their experiences.

Finally, there is the question about the suitability of the selected analysis method. To accomplish a more robust categorization of the dimensions isolated during the phenomenological analysis, the first and second author carried out a categorization process of the reports. Moreover, two external researchers who were not involved in the acquisition and preparation of data, nor the first steps of the analysis, coded the reports analyzed by assigning them a category from the categories isolated by AA-S and ED. However, the resulting scores from the intercoder agreement were not very high, showing that in most cases, the external researchers would have coded the descriptions differently, either by classifying them with a different category, or by naming the category differently. This resulted in the modification of some categories and the re-assignation of some descriptions to the most appropriate category.

The lower score obtained in the intercoder agreement calls into question the rigor of the categorization process undertaken in the analysis of the results. For instance, dream researchers utilizing validated scales for examining content analysis (see Hall and Van de Castle, 1966) advocate for high scores in intercoder agreement for the validation of the results (see Schredl et al., 2004). However, some studies have pointed out the significant differences between self and external ratings of dream reports (i.e., between self-ratings undertook by the participants and ratings carried out by external judges) and how judges tend to underrate some elements of the reports (see Sikka et al., 2014). Similarly, this problem occurs in studies like ours undertaking phenomenological analysis. Given that the categorization process results from a bottom-down process, from the analysis of the reports to the creation of categories, the resulted categories tend to be in a high level of abstraction. Moreover, these categories are oftentimes context-sensitive, and as such, some dimensions (i.e., “Sense of self”) might be very difficult for an external researcher to properly classify without further background involvement in the interview process. As we showed in section Synchronic Categories, while some participants might have mentioned that their experience was “bodiless” or “selfless,” other descriptions by the same participant provided further details on the different aspects of their self-experience. Thus, there is an extent to which the categories isolated are given meaning through engagement in the understanding of participant’s answers, which requires a certain level of interpretation by the coder (Krippendorff, 2004). Moreover, the external coders were not given the entire interview, but only selected excerpts. Nevertheless, we consider that the external coding was a beneficial step in our analysis process, which identified categories that needed revision and adjustment, but also those that seemed to work quite well and that should then be accounted for in future research. Finally, it should be noted that the categories isolated refer to the sample of reports analyzed, and that for the creation of more robust categories, or even for the possible creation of validated scales involving such categories, a larger sample would be needed.

## Conclusion

This paper presented the results of the second stage of the research study on “*Objectless sleep experiences*” which aimed at investigating possible instances of awareness had during sleep in absence of a distinct object of awareness. To that end, we carried out 21 extensive phenomenological interviews describing an occurrence of what participants took to be a sort of “objectless state” during sleep. From the phenomenological analysis, we distinguished a common phase across 12 of those participants that we coined “nothingness phase.” Moreover, the analysis yielded the emergence of 6 experiential categories with their corresponding second and third-level categories characterizing the described phase of nothingness as lacking an explicit bodily awareness, yet with a minimal sense of ownership within the experience, as well as a minimal sense of first-person perspective and the feeling of being in an indeterminate location. This phase was also characterized by the presence of a sense of agency, with an agent that in most cases reported a lack of control of the situation. There were also frequent descriptions of modality-like sensations, such as feeling “sound” or “visual percepts” in absence of either hearing or vision. Most participants also described the presence of positive or fairly positive emotions. While initially, participants reported to have experienced a state in which there was nothing to be aware of other than the fact that they were aware, our interview protocol unveiled different aspects of such a state involving some contents of awareness. The results presented here add valuable data on how we should characterize states of awareness that are said to be objectless—instances in which one says to be aware of nothing.

## Data Availability Statement

The raw data supporting the conclusions of this article will be made available by the authors upon request, as long as it does not compromise participant’s privacy as signed in the Ethics form.

## Ethics Statement

The studies involving human participants were reviewed and approved by the College of Arts, University of Glasgow. The participants provided their written informed consent to participate in this study.

## Author Contributions

All authors have made a direct, contribution to the present work. AA-S conceived the study, secured funding, prepared the data for analysis, executed the final analysis, wrote the first draft of the manuscript, prepared the last version, and created the diagrams and figures. AA-S and ED designed the study protocol, recruited participants, carried out interviews, and undertook the initial phenomenological analysis. ED prepared the interviews’ verbatim. TC-F and SGT-P undertook the external coding of the interviews. ED, TC-F, and SGT-P provided the substantial feedback and comments on the subsequent versions of the manuscript. All authors contributed to the article and approved the submitted version.

## Funding

AA-S was supported by the Scottish Graduate School for Arts and Humanities (SGSAH) Doctoral Training Partnership (DTP; grant number AH/R012717/1) and a grant by the International Association for the Study of Dreams (IASD) and the Dream Science Foundation (DSF).

## Conflict of Interest

The authors declare that the research was conducted in the absence of any commercial or financial relationships that could be construed as a potential conflict of interest.

## Publisher’s Note

All claims expressed in this article are solely those of the authors and do not necessarily represent those of their affiliated organizations, or those of the publisher, the editors and the reviewers. Any product that may be evaluated in this article, or claim that may be made by its manufacturer, is not guaranteed or endorsed by the publisher.

## Footnotes

^1^The state of ‘sushupti’ is mentioned in the Mandukya Upanishad and the *Yoga Sutras of Patañjali.* For a more detailed account of sushupti in these texts, see the translations and commentaries by Gambhirananda (1937), Nikhilananda (1949), and Olivelle (1998) (for the Mandukya), and Arya (1986), Aranya (1989), and Bryant (2009) (for the Yoga Sutras).

^2^A detailed account of the answers given to the survey for each question and the rationale on which of those questions alluded to the targeted experience will be provided in a separate paper (Alcaraz-Sánchez, *under preparation*).

^3^A full description of demographics and answers provided in the survey by those participants can be found in **Supplementary Material**.

^4^This subjective aspect of the experience is also called the “subjective character of the experience,” or the “what-it-is-likeness” of the experience in philosophy (Nagel, 1974).

^5^Such a view of non-dual awareness is supported by reflexive theories of consciousness (or self-illumination theories), which hold that when we are aware, we are necessarily pre-reflectively or tacitly aware of our own awareness (see MacKenzie, 2008 for a discussion). Reflexivist views on consciousness are found in certain schools of Indian, Chinese and Tibetan Buddhist philosophy, but are also defended by the phenomenological tradition (see Thompson, 2011). Note that it is a contentious point of debate in analytic philosophy of mind as to whether such states would involve second-order representations, and whether such states should not be better understood as self-representational (see Kriegel, 2019 for a discussion).
