# Supplementary material for: Nothingness Is All There Is: An Exploration of Objectless Awareness During Sleep
Source: Front Psychol. 2022 Jun 10;13:901031. doi: 10.3389/fpsyg.2022.901031 (PMC9226678; doi:10.3389/fpsyg.2022.901031)
Supplement: Supplementary file 1 [file Data_Sheet_1.DOCX]

**Interview sessions**

# Examples of interview questions

The interviews conducted weren’t based on a semi-structured interview template, but instead, the questions made were formulated in accordance with the responses gathered by the participants. However, as per the micro-phenomenological interview method (MPI), some examples are given about leading questions that can be asked in the different phases of the description

## Setting up

**Interviewer:** *I will ask you some questions about the experience that you choose to talk about for this interview, by focusing on how it felt to be in that particular experience. I will guide you through the process of remembering that experience and I will help you explore different dimensions of it. Sometimes I will ask you for some clarifications, but some others I will move to a different dimension of the experience. I will also rephrase some of the things you say, but feel free to interrupt me if I rephrase them wrong. It can be that when you hear me saying back what you just said you feel that something isn’t right, or that you didn’t mean that. That’s totally fine. Also, remember that you aren’t obliged to answer all the questions and we can stop anytime you wish, just let me know.*

*For this interview we asked you to think about an experience that you have had recently while sleeping in which you recall some sort of awareness in absence of dreams. It’s important that you pick a specific experience. Doesn’t matter if it’s too short, the important thing is that you can recall it very vividly. You can take some moments to think about it if you wish.*

## Beginning of the experience: situating the participant

**Interviewer:** *Ok [participant’s name], let’s go back to the beginning of the experience, when [reformulation of their description] happened. How did started?*

From this point, different questions are made to explore different components (these are adjusted in base of the answers provided)

- **Visual components:** *Do you see anything? I would like you to describe the place. Look around and what do you see?*
- **Auditory components**: *At this moment, do you hear anything? Listen again, find noises. What is its volume? Its tone? Is it your own voice, or the voice of somebody else?*
- **Kinaesthetic or interoceptive elements**: *What is the position of your body? Do you feel anything? What do you feel? Is this feeling located somewhere?*

*What do you feel? Is this feeling located somewhere?*

- If **nothing mentioned**: *When you do nothing, what do you do? Is there anything else? What else is going on at this time? Did you have perhaps feelings, thoughts or anything else at this moment? What else is happening at this moment?*

## Deepening into the experience

Again, the interviewer picks one of the dimensions to explore and guides the participant through the different elements of it. As before, different questions are asked based on the answers provided.

- *And when you do X, what do you do?*
- *And when you feel X, what do you feel?*
- *And when you are aware of X, what do you do?*
- *Does this feeling/attention/awareness have a shape/ texture/ size? What is it like?*
- *Does this feeling/attention/awareness have a direction?*

# Post-interview questions

Below are the questions asked after each interview phase; for assessing the memory recall of the experience of spelling a word and memory recall of the experience of objectless dreamless sleep chosen.

- **Vividness**: If you were to evaluate the level of vividness you had while recalling the experience, from 0 to 10, which would be?
- **Completeness**: And, how complete was the recollection? Did you feel something missing? Again, from 0 to 10.
- **Invention**: To what (if any) extent did you feel, while describing your experience, that you were constructing or inventing what ended up being described? (0 none, 10 a lot)
- **Articulation**: How well did / do you feel that you managed to articulate the described experience within the interview?

# Excerpts

We have included two short excerpts from two interviews to illustrate the application of the phenomenological method carried out. The first excerpt provides a good illustration of questions made by following the micro-phenomenological method to explore different aspects of the experience, inviting the participant to focus on the subjective character of the feelings and sensations had. The second excerpt exemplifies the sort of questions attempted when a participant is describing a state that apparently lacks any feelings and they are finding difficult how to articulate the experience had.

For each transcript provided, participant’s answers were highlighted in bold. The speech in brackets denotes a verbalisation made at the same time, or immediately after, someone’s else. The speech in capital letters and underlined denotes a stress in intonation.

## Excerpt 1

**P#07: Yes, actually, now I remember that I wasn't any more in this 3D inner space. But I was in this factory setting then. [**Mhm] **And I was even less aware of my real-world surroundings then. It was actually quite like a dream but without the visual content...**

**Interviewer:** So there is nothing that you SEE in this moment when you're in the factory space?

**P#07: Not like seeing with my EYES.**

**I:** But, and so if you're in this moment, you suddenly feel you don't know how the transition happens, but you suddenly feel like you are in this factory space. And, do you feel INSIDE the space? Is the space bigger than you? **[Yes]** And you feel it from your first-person kind of position of your body? **[Yes]** Are you aware of the SPACE? And of your body in the space?

**P#07: No, I actually didn't have the BODY in this space. It wasn't just my awareness that was there.**

**I:** But it feels like YOU? It, does it feel like you're looking at it from the outside, or looking at it from the inside...?

**P#07: Well, I was INSIDE this BUILDING, but I couldn't see from the outside or anything like that, standing in front of this room, or BEING in front of it. Because it well, it didn't feel like I was in the normal body.**

**I:** So it feels more like a point of awareness or point of perception? **[Yes, but not that single-pointed. More like this cloudy shape that we had earlier]**

**I:** Mm hmm. Okay. And is it... By the way, is this like a familiar space? This factory building?

**P#07: No. Not at all.**

**I:** So when you're there, you're saying that you're not seeing anything in the sense that you would see with your eyes? And how do you know that you are in a factory space? And how do you know that you're in front of a sliding door?

**P#07: For once, I can FEEL it. And...** 5' **It's this spatial representation again, that's that I'm aware of.**

**I:** So you can feel it and there's a spatial representation of it. But could you imagine for a moment that I have no idea what spatial representation means? And just try to describe to me if I was in your shoes, or if I was this cloudy awareness in that moment? How, how would I feel the sliding door? Or how good would it be for me to have this spatial representation?

**P#07: It's like, I could feel the DISTANCE from my awareness in there, to the walls and to the door, and so on. And, yes...** 3' **there are different points that make up this space. And I can feel those inside of me. *[Laughs]***

**I:** So it feels like there IS space and that this awareness or YOU or however we call it has a certain LOCATION within that space.

**P#07: Yes, but it's not that FIXED. It can... It moves around a bit, but the surrounding stays the same. Even if it's not that fixed like something in the real world.**

**I:** Mm hmm. And just a very technical question, but is there a relative distance from this moving awareness to the walls and to the door, right?

**P#07: Yes. And (inaudible) [*Laughs*.]**

**I:** And... is awareness moving around?

**P#07: Not all the time, but it can change... its place**

And if it is distant, if there is a certain distance from the sliding door, for instance, is there also a certain level, like a certain height on which this awareness is?

**P#07: Yes, that's, that's like I would experience it normally, being in a body walking there**

**I:** Mm hmm. But you, you know that you are NOT in a body, you don't FEEL in a body?

**P#07: No, it didn't feel like that. I couldn't SEE it or something like that. It just didn't FEEL to be there. Because in that state, I wasn't aware of my body at all.**

**I:** Was there anything? So, in the, when you are IN this experience, Is there anything strange about that? Does it feel in any way strange or different that you are NOT in your body?

**P#07: No. [*Laughs*.] I didn't even notice that until we spoke about it.**

**I:** Mhm. Okay, so you are standing in front of this sliding door, and you don't really SEE it. So, it's not a visual thing. So, you... Is there anything visual to the experience at all? Like any colour or brightness, darkness?

**P#07: Um, there were no colours. But there's TEXTURE. It's a wooden door.** 5' **But I can't say about the walls, they were not exactly like normal walls.** 4' **They were just... the limits... for my awareness in this place, maybe...**

**I:** Is there a size to this to this factory building? Like how tall are the ceilings? Or how wide the walls?

**P#07: This place I was in was like a pretty normal room size.**

**I:** And do you have any idea how you know that it's a factory building?

**P#07: I think it's just this sliding door, on this... this room I was in, that reminded me of it. Because I'm not aware of the building.**

**I:** So there is no... nothing that you see. But you know that there is a sliding wooden door, and it has a wooden texture. And you are aware of this FROM a certain height, similar to the height that you would have if you were there in your body. But you are paying attention or you're aware of that from a kind of like cloudy type of awareness that sometimes moving and but sometimes static?

**P#07: Yes. *[Excited]* I think it moves when... I thought about something else and wasn't directly IN this experience, but maybe thought about what it means or what I should do now or something like that. And if I then returned with my awareness to the experience, then it may have changed the place.**

## Excerpt 2

**P#08: It all kind of went into a more chaotic state in the... that was very uniform. The next state just becomes a blur. There's not much going on, I don't have the narcotic feeling anymore. I'm not having much of any feeling. There's just some, some visual patterns moving from upper left to lower right. And maybe towards centre a little bit that I would think of is like, a little bit like seeing sleep outside a window, you know, where there's impression that something's moving into that pattern. And there wasn't much else going on. That just went on for a while. It's kind of like, nothing, it's closer to that white state where there wasn't much of thought or emotion or anything, or feeling. There was still some background imagery. Not very interesting. I don't know how long it went on.** **There's probably some part of me that thought it is kind of pretty interesting, but it's not, not interesting when you've been doing it for decades, just, just I was there. And there was a slight pattern of streaking. And that I don't know how long that went on. Because that was also like, very little temporal sense. And then that went into the pure white, so...**

I: So how do you feel this transition from this state? Is a bit more chaotic and not that interesting to the, the pure white one, plain white one…?

**P#08:** **Yeah, that is almost impossible to describe. I'm not sure how you're gonna deal with it. There is an awareness you're there. Like, I was, I was there. I was still lucid, but my thought process stopped. So I think it's like when they talk about meditation, one of the meditation types. They try to stop thinking to have a silent mind. Which, oddly I can't really achieve in daily life. After decades, that's one of the hardest meditations. But in this state, yeah, the thought process just stops. There's just white, it's not even a feeling so much of it being directly in front of me. It's just white. For lack of anything else, I mean, it's not black. It's...** 7' **I guess, the way I look at it is maybe black would be there's no energy or whatever. And this is full of energy, although that's more esoteric. It's, it's, I don't know why it's white. But it also doesn't involve the feeling and the emotion, or the sense of motion. Those things that I just described. It's just being there... But nothing's going on.**

**I:** And when you say that it is just white in opposition to it's not black, is it that visual? So, it's like, now was like, I don't know, like, kind of when starring at a light bulb or something, and you see white? Would that be like that, or…?

**P#08: Yeah. It's like, if, in one, you're looking at a movie screen that has nothing on it. And in other, someone turns out, light.**

**I:** I see, okay…

**P#08:** **This is like a movie screen with nothing on it. There. It's not black. But there's nothing there. So... I think that's why they call it clear light, because it's hard to describe. It's the lack of anything, including black.**

I: But it has white on it. So, then it's an experience of... [**Yeah, I would have to relate it to white, I mean, it's, it's, at this point, you're getting so deep into weird stuff that it's like trying to describe... I don't know, like when they say describe taste to someone, if you can't use any descriptive [inaudible], and they can't compare it to anything that involves taste. So what I would say is, it's... it is the lack of anything, including black. And the way my brain interprets that is white.**

**I:** Now, that's okay, when I'm asking you some questions, and I am very aware that this might be difficult to describe, I'm asking you to think about which things are not, no? So, in a way, like you said, it's not black, it's not like seeing something that is white in front of you. So, we know it's like the lack of anything. And it was like if it was light... [**Yeah, it's kind of like seeing white in front of me. That's not exactly what's going on. But that's the closest I can get to describing it]**

**I:** Okay. And then you're in that stage, which is different from the one that you were coming from, which was the one it was that was a bit more chaotic, and that there were these still these images or some things like moving around, that we didn't know how long it lasted. And now you have moved to this one, which the thoughts have disappeared. There's nothing there. And it's only whiteness, there's no feeling, no sense of motion. Are you aware of them? In this state?

**P#08**: **I am aware that not in the traditional sense. Traditional sense would have to involve some thing you can relate to, right? Time or thought. You know, you're definitely crossing into esoteric thought here where... there is... you're there, there's an awareness. But there's nothing to be aware of... So, I wish I could, I wish I could just hand you an answer here.**
