# Supplementary material for: Nothingness Is All There Is: An Exploration of Objectless Awareness During Sleep
Source: Front Psychol. 2022 Jun 10;13:901031. doi: 10.3389/fpsyg.2022.901031 (PMC9226678; doi:10.3389/fpsyg.2022.901031)
Supplement: Supplementary file 2 [file Data_Sheet_2.DOCX]

**Full list of categories isolated with descriptions**

# Sense of self (SS):

| This higher-order category involves different second-order categories that refer to how the feeling of being someone—or a sense of ego—is instantiated. The second-order categories have been constructed from features that appear repeatedly in the reports, but they are also informed from the different dimensions proposed in the literature as the basis of the sense of self. |
| --- |

## 1A. Body ownership:

| This dimension refers to the feeling of having a body or identifying oneself to be within some set of boundaries (even if those aren’t within a normal body). It ranges from a more explicit sense of body ownership that might involve mentions to bodily feelings, body parts or having a body, to mentions of lacking a body, yet having some minimal sense of self-identification within the experience. |
| --- |

### Body parts/bodily processes (SS1A1)

| Descriptions that allude to the sense of having or owning a body. They might mention the body or body parts and/or the location of a body in relation to space (including body position) or bodily processes (such as breathing). |
| --- |

### Weak embodiment/lack form (SS1A2)

| Descriptions that allude to an identification with a body, yet it involves a very weak sense of embodiment or description of a body that lacks a form. Many descriptions involve an explicit mention to the fact that part of the attention was on one’s own body, yet one didn’t feel it strongly—one knows that one had a body without being explicitly aware of it. |
| --- |

### Distorted (SS1A3)

| Descriptions that describe having a body in an odd or impossible position or feeling oneself as being a distorted sort of body, like a cloudy shape or cloud of energy. |
| --- |

### Minimal identification (SS1A4)

| Descriptions that mention the fact that one has lost one’s body. In most cases, these descriptions are still accompanied by reports that include some sort of minimal sense of self-identification, either with a body part or something that isn’t oneself (‘the void’, ‘a speck’). It also includes more esoteric-sounding or unconventional descriptions such as the feeling of a particular sensation (‘a sound’, ‘a pulsing’, ‘a ripple’) to describe the way in which oneself was part in the experience. |
| --- |

## 1B. Spatial self-location:

| This dimension refers to the feeling of being located somewhere (a sense of ‘here’). It can be more explicit or more minimal, ranging from mentions of oneself being in a specific point in space, a sense of relative location to space to the feeling of just being ‘there’. |
| --- |

### Physical (SS1B1)

| Descriptions mentioning a *self* (or an ‘I’) who is located in a point in space, usually one that have some physical or spatial features (regardless of if the actual environment is in fact located in the physical world). There’s a feeling of being located somewhere in reference to that space, including a sense of relation towards the space which is recognised as either the actual sleeping environment or the dream world. |
| --- |

### Fluctuating (SS1B2)

| Descriptions similar to the previous, yet involving mentions of having been in two locations at the same time, such as oneself feeling located in one point, but with a perspective experienced from a different point. |
| --- |

### Indeterminate (SS1B3)

| Descriptions that appear to be similar to the previous sub-category, inasmuch as they involve a clear self/other distinction (an ‘I’ or a ‘self’ distinct to something else). They might still involve a sense of position relative to space, yet one doesn’t know where that is, or they might lack any explicit mention of being in a particular point in space, other than the feeling of being in relation to ‘something’ else outside oneself. |
| --- |

### Minimal (SS1B4)

| Descriptions saying to lack a sense of self-location other than a sense of being ‘there’ in the experience. It might include allusions to the fact that one is ‘nowhere’ or that whilst the experience started by one feeling to be located somewhere, it has now transitioned to a state where one merely feels to be ‘in the state’ but without a clear sense of self-location. |
| --- |

### No clear boundaries (SS1B5)

| Descriptions that only involve a subjective perspective as location – the experience is lived from an egocentric point of view, yet this might not involve a clear sense of boundaries between oneself and the other. Some descriptions allude to a sense of being so immersed with the experience that they can’t distinguish themselves from it. |
| --- |

### Absent (SS1B6)

| These descriptions involve explicit mentions to the fact that they one didn’t feel oneself as having been ‘*in’* the experience while it was unfolding, even lacking a minimal sense of ‘being there’. |
| --- |

## 1C. Perspective:

| This dimension refers to the point of view of the ‘self’ or the ‘I’. In some cases, the same description applies to both ‘Perspective’ and ‘Spatial self-location’. The difference between both is that ‘Perspective’ refers to the point from which the experience is lived (which is usually an egocentric point of view) whereas ‘Spatial self-location’ refers to a feeling of being in ‘there’. |
| --- |

### Regular (SS1C1)

| Descriptions involving a first-person experiencing something that is outside oneself, closer to ordinary wakefulness experience. Egocentric point of view. |
| --- |

### **Fluctuating (SS1C2)**

| Descriptions of an egocentric point of view, that might involve two locations or more at the same time, such as seeing oneself from outside or having multiple viewpoints at the same time |
| --- |

### Minimal (SS1C3)

| Descriptions that prima facie doesn’t seem to involve an ordinary subject or experiencer, other than a minimal point of view from which the experience is had. They usually come together with descriptions of lacking a sense of body ownership or lacking a sense of being in a specific spatial point in the experience. In some cases, descriptions might allude to a sense in which one was being unable to say where one’s perspective was, since they are one with the environment. Other descriptions involve a sense of just ‘observing’ or ‘watching’ the experience. |
| --- |

### Absent (SS1C4)

| Descriptions that don’t seem to involve a sense of being oneself having the experience, or any sort of egocentric point of view whilst the experience was unfolding. |
| --- |

## 1D. Agency and attitude

| This dimension includes reference to different aspects of a sense of agency, which can be more explicit, like mentioning intentions one had during the experience, motivations or actions initiated (or wanted to initiate), but also references to the attitude taken towards the experience, such as letting it be, or accepting it. It also includes mentions to having lost the control of what is happening or being unable to take control. |
| --- |

### **Active** (SS1D1)

| Descriptions alluding to an active agent that wants to take some sort of action towards the experience. These descriptions might include a sense of wanting to stay longer in the experience, to explore the state, to pay or maintain their attention or to change something about the experience. |
| --- |

### **Receptive (SS1D2)**

| Descriptions that still involve a sense of agency, although a more ‘passive’ or acceptant sense of agency. They mention the fact that one could initiate action but one decided not to take it, or that the action chose was to take a more passive role in the experience (i.e. observing, letting it go). Some descriptions allude to how one was actively seeking to ‘focus on not focusing’, to not disrupt the unfolding of the experience. |
| --- |

### Lost control/out of control (SS1D3)

| Descriptions explicitly mentioning how something occurs that holds or forces oneself from doing something. |
| --- |

# **Sensations (SE)**

| This higher order category involves descriptions of different sort of sensations had by the participants, classified into three kinds (or sub-categories): 2A.Bodily sensations, 2B. Kinaesthetic sensations and 2C. Non-modal sensations. |
| --- |

## 2A. Bodily sensations

| This second order category includes descriptions of sensations that are located in the body or refer to bodily feelings. These descriptions might allude to sensations on a body part or the feeling of a body part (including bodily processes such as breathing). Descriptions here are quite resemblant to actual bodily sensations had during wakefulness. |
| --- |

### Breathing (SE2A1)

| Feeling or sensation that one is breathing, including body parts involved (i.e., inside of the nose) |
| --- |

### Tactile sensations (SE2A2)

| The feeling of touch, including the feeling of body in contact with an object |
| --- |

### Temperature (SE2A3)

| Feeling of temperature, including coldness or heat. Here, we code those descriptions mentioning the actual feeling of temperature on the body or a body part, and not those describing the features of a particular feeling with allusion to temperature-related adjectives. |
| --- |

### Absent (SE2A4)

| Explicit mentions to the fact that one isn’t having physically like (or bodily like) sensations during the state. |
| --- |

## 2B. Kinaesthetic sensations

| This second order category involves more proprioceptive sensations, which they can be related to the body (it might involve the position of the body or an implicit sense of having a body), yet not necessarily. |
| --- |

### Position (SE2B1)

| Feeling one’s body to be in a certain position. |
| --- |

### Motion/Gravity (SE2B2)

| Feeling oneself moving, with or without reference to one’s body. It might involve different sort of motions, such as going down, going forward, or just a sense of gravity. |
| --- |

### Floating/hanging or suspended in the air (SE2B3)

| Similar to the previous, but in this case there’s a sense of feeling lack of gravity, or feeling that one is suspended or floating in the air |
| --- |

### Release tension/relief (SE2B4)

| Descriptions that allude to feeling how the tension is released, and thus, one is now felt more relieved. It might include allusions to the body or not |
| --- |

### A force/barrier (SE2B5)

| Descriptions that allude to feeling some sort of force or energy. This might be something that holds oneself back, or it can be just a way to describe a change in the experience |
| --- |

### Absent (SE2B6)

| Explicit mentions to the fact that one isn’t feeling anything, including any proprioceptive sensations |
| --- |

## 2C. Non modal sensations

| This second order category involves descriptions of different sensations had in relation to a state that lacks any bodily or kinaesthetic sensations. These are described as ‘non-modal’ sensations since they don’t seem to belong to any particular sensorial modality (i.e. sight, hearing, touch etc) or to any sensation had within the body. These descriptions allude to how the ‘emptiness’ or the ‘nothingness’ is felt. |
| --- |

### Modality-like (SE2C1)

| Descriptions alluding to one sensorial modality, yet oneself stresses how the experience didn’t involve such a sensorial modality (i.e. hearing, seeing or touch without hearing, seeing or touching). |
| --- |

### As having material properties (SE2C2)

| Descriptions that allude to the ‘nothingness’ as ‘something’, as a ‘thing’—like an object that has material or physical properties. |
| --- |

### As lacking anything (SE2C3)

| Descriptions that allude to feeling the ‘nothingness’, not as a thing, but as a sensation of ‘nothingness’. This sub-category involves descriptions that attempt to describe how is it like to feel nothing. |
| --- |

# **Visual experience** (VE)

| This category involves descriptions about the type of visual experience had or the lack thereof. Note that the descriptions refer to the content of the visual experience (what was seen or perceived) or how things were seeing (quality of the vision or vividness of the content of the visual experience). |
| --- |

### Simple imagery (VE1)

| Descriptions of very simply object-like imagery, including simple mathematical, or geometrical forms, but also texture-like and pattern shapes. |
| --- |

### Gradual loss of imagery (VE2)

| Descriptions mentioning the gradual dissolution or fading of the visual experience, including seeing blurry or dim. |
| --- |

### Loss of imagery (VE3)

| Descriptions that allude to the lack of any imagery, yet it contains mentions to some visual elements such as colour, light or absence thereof. |
| --- |

### Absent (VE4)

| Specific mentions to the lack of a visual sense, different from perceiving darkness (or any other colour) or lack of imagery. |
| --- |

# Emotion (EM)

| This category refers to the emotional component of the experiences reported. It involves descriptions of emotions or feelings had. |
| --- |

## 4A. Emotional sensations

| Descriptions referring to the presence or absence of emotions and feelings had during the experience. |
| --- |

### Presence (EM4A1)

### **Absence** (EM4A2)

# Attention (AT)

| This category involves descriptions about how one’s attention was—the type of attention |
| --- |

### Focused (AT1)

| Descriptions of an attention that is focused on specific content of awareness, or aspects of the perceptual experience. Examples might include descriptions of thoughts, intentions, or what was imagined or visualised |
| --- |

### Dynamic (AT2)

| Descriptions of an attention that fluctuates towards different dimensions of the experience, or an attention that they feel they can manipulate |
| --- |

### Resting/Vague (AT3)

| Descriptions that mention the fact that there wasn’t any explicit focus of attention, yet one was trying to focus on not focusing or one was just observing the experience. |
| --- |

### Wide attention, no focus (AT4)

| Descriptions that also mention not having an explicit focus of attention, but more of a sense of just being aware, with nothing to be aware of or pay attention to. |
| --- |

# Awareness of the state (AS)

| This category involves descriptions of what one takes one’s experience to be and the sort of awareness had of that fact. It might involve a more explicit or implicit awareness. |
| --- |

### Knowing they are asleep:

| Taking that one is sleeping, that the state is one of sleep. This might be just an implicit sense of knowing that one is sleeping, without conscious thoughts about that fact (such as the thought of ‘I am sleeping right now’) |
| --- |

### Knowing they are in bed

| Taking that one is in bed. In some cases, it might come together with the awareness that they one is asleep. Descriptions can be more or less explicit about the fact that one is in bed, and it might just involve a sense of knowing that one is in bed without paying attention to that fact. |
| --- |

### Knowing they are dreaming

| Taking that one is dreaming. It can come in different degrees. |
| --- |

### Knowing they are aware

| Taking that one is aware. Descriptions here might involve a sense in which one is aware of being aware or one describes how one’s awareness was (it might be related to the previous higher-order category ‘Attention’). |
| --- |
