# Supplementary material for: Nothingness Is All There Is: An Exploration of Objectless Awareness During Sleep
Source: Front Psychol. 2022 Jun 10;13:901031. doi: 10.3389/fpsyg.2022.901031 (PMC9226678; doi:10.3389/fpsyg.2022.901031)
Supplement: Supplementary file 3 [file Data_Sheet_3.DOCX]

Quantitative analyses

# Demographic information

The following table provides a summary of the demographics (including lifestyle choices, health and interest in dreams) of those participants (n=12) whose experiences were selected for the phenomenological analysis. The results are obtained from the answers given by those participants to the online survey ‘Objectless sleep experiences’ which was the first stage of the present research project, and which was used for shortlisting participants for the interview stage.

| Participant’s demographical information (n=12) | |  |
| --- | --- | --- |
| General demographics | |  |
| Age | *mean* |  |
| *(Years old)* | 36.5 |  |
| Gender | *% (count)* |  |
| Female | 42% (5) |  |
| Male | 58% (7) |  |
| Country of residence | %(count) |  |
| Canada | 8% (1) |  |
| Finland | 8% (1) |  |
| Germany | 17% (2) |  |
| Slovenia | 8% (1) |  |
| US | 58% (7) |  |
| Native language | *%(count)* |  |
| English | 67% (8) |  |
| Finnish | 8% (1) |  |
| German | 17% (2) |  |
| Slovenian | 8% (1) |  |
| Physical and mental health |  |  |
| Physical condition | *%(count)* |  |
| No | 92% (11) |  |
| Yes | 8%(1) |  |
| Mental health | *%(count)* |  |
| No | 75% (9) |  |
| Yes | 25% (3) |  |
| Currently taking medication that affects CNS | *%(count)* |  |
| No | 75% (9) |  |
| Yes | 25% (3)  (Antidepressants) |  |
| Sleep Indicator Score (SCI) | Mean* |  |
| *(Out of 32)* | 27 |  |
| *(Out of 10)* | 8.7 |  |
| *Mean scores lower than 16 indicate a possible insomnia disorder (see Espie et al. 2014) |  |  |
|  |  |  |
|  | |  |
| Religion | |  |
| Religion adhesion | %(count) |  |
| Buddhist | 17% (2) |  |
| Christian | 8% (1) |  |
| None | 17% (2) |  |
| Other | 17% (2) |  |
| Spiritual but not religious | 42% (5) |  |
| Importance religion | Mean |  |
| *(Out of 100)* | 63.1 |  |
| Alcohol intake |  |  |
| Alcohol consumption | %(count) |  |
| No | 33% (4) |  |
| Yes | 67% (8) |  |
| Frequency | %(count) |  |
| Between 1-2 units a week | 25% (3) |  |
| Between 2-3 units a week | 8% (1) |  |
| Less than 1 unit | 25% (3) |  |
| Over 4 units a week | 8% (1) |  |
| Recreational drugs intake |  |  |
| Regular recreational drug consumption | %(count) |  |
| No | 8% (1) |  |
| Yes | 42% (5) |  |
| Type of drugs consumed | % (count*)  *Multiple answers allowed |  |
| Cannabis | 16.6% (4) |  |
| Psychedelics (including ayahuasca and DMT) | 8.3% (2) |  |
| Frequency of drug intake | %(count) |  |
| Every day | 8% (1) |  |
| Once a month | 8% (1) |  |
| Twice a month | 8% (1) |  |
| Twice a week | 17% (2) |  |

| Meditation practise | | | %(count) |  |
| --- | --- | --- | --- | --- |
| No | | | 8% (1) |  |
| Yes | | | 92% (11) |  |
| Frequency of meditation practise | | | %(count) |  |
| Every day | | 50% (6) | |  |
| Every other day | 17% (2) | | |  |
| Less than five times a month | 8% (1) | | |  |
| Less than twice a week | 17% (2) | | |  |
| Style of meditation practised | | | %(count*)  *Multiple answers allowed |  |
| Mindfulness | | | 11.1% (4) |  |
| Shamata | | | 2.78 % (1) |  |
| Transcendental meditation (TM) | | | 2.78 % (1) |  |
| Zen | | | 2.78 % (1) |  |
| Other: Kundalini, Yoga Nidra,  “Breathing relaxation”,  “Specific instructions given by Lama”, Zen Shikantaza and “Narrow focus meditation”,  “Guided third eye meditation”,  “Creative visualisations” | | | 22.22 % (8) |  |
| Frequency meditation | | | % (count) |  |
| Between 15-30minutes | | | 67% (8) |  |
| Less than 15minutes | | | 8% (1) |  |
| Over 30minutes | | | 17% (2) |  |
| IMPORTANCE MEDITATION | | | mean |  |
| (Out of 100) | | | 83 |  |

# Self-ratings

The table below provides mean values for the self-ratings across all participants (n=12) for each of the dimensions of the experience of recollecting the spelling word (first-part interview; vividness1, recollection1, invention1, articulation1) and for the experience of recollecting an instance of contentless awareness during sleep (second-part interview; vividness2, recollection2, invention2, articulation2). Each dimension was self-rated from 0-10 (0 low and 10 high).

| Vividness1 | Recollection1 | Invention1 | Articulation1 | Vivivdness2 | Recollection2 | Invention2 | Articulation 2 |
| --- | --- | --- | --- | --- | --- | --- | --- |
| 8.4 | 9 | 1.8 | 7.1 | 8.9 | 8.6 | 1.1 | 6.9 |

# Intercoder agreement

Fleiss Kappa’s values for the intercoder agreement were computed across the coding given to the selected descriptions in the reports by the different coders (Coder 1 and Coder 2= External coders; Coder 3= Main investigator).

| Descriptions= 202 | Across coders (Coder.1, Coder.2 and Coder 3) | Coder.1 and Coder.2 | Coder.1 and Coder.3 | Coder.2 and Coder.3 |
| --- | --- | --- | --- | --- |
| Kappa* | 0.481 | 0.357 | 0.627 | 0.458 |
| Z | 67.7 | 29.2 | 50.2 | 36.1 |

*Kappa’s coefficient values are interpreted following Fleiss et al. (2003) who suggest the following interpretation: **>0.75:** Excellent agreement beyond chance; **0.40-0.75:** fair to a good agreement beyond chance; **<0.40:** poor agreement beyond chance.

Fleiss Kappa’s values for the intercoder agreement for all categories across all coders.

|  | Kappa | Z | p.value |
| --- | --- | --- | --- |
| AS1 | 0.436 | 10.724 | 0 |
| AS2 | 0.620 | 15.262 | 0 |
| AS3 | 0.738 | 19.267 | 0 |
| AS4 | 0.551 | 13.556 | 0 |
| AT1 | 0.113 | 2.789 | 0.005 |
| AT2 | 0.158 | 3.898 | 0 |
| AT3 | 0.521 | 12.838 | 0 |
| AT4 | 0.316 | 7.789 | 0 |
| EM4A1 | 0.797 | 19.612 | 0 |
| EM4A2 | 0.855 | 21.059 | 0 |
| EM4B2 | 0.245 | 6.032 | 0 |
| SE2A1 | 0.422 | 10.386 | 0 |
| SE2A2 | 0.390 | 9.599 | 0 |
| SE2A3 | 0.663 | 16.329 | 0 |
| SE2A4 | 0.708 | 17.417 | 0 |
| SE2B1 | 0.898 | 22.114 | 0 |
| SE2B2 | 0.663 | 16.329 | 0 |
| SE2B3 | 0.663 | 16.329 | 0 |
| SE2B4 | 0.498 | 12.268 | 0 |
| SE2B5 | 0.493 | 4.760 | 0 |
| SE2B6 | 0.660 | 8.124 | 0 |
| SE2C1 | 0.679 | 16.703 | 0 |
| SE2C2 | 0.395 | 9.724 | 0 |
| SE2C3 | 0.390 | 9.599 | 0 |
| SS1A1 | 0.436 | 10.735 | 0 |
| SS1A2 | 0.240 | 5.907 | 0 |
| SS1A3 | 0.240 | 5.182 | 0 |
| SS1A4 | 0.348 | 8.566 | 0 |
| SS1B1 | 0.575 | 14.153 | 0 |
| SS1B2 | -0.008 | -0.205 | 0.838 |
| SS1B3 | 0.256 | 6.294 | 0 |
| SS1B4 | 0.269 | 6.618 | 0 |
| SS1B5 | 0.113 | 2.789 | 0.005 |
| SS1B6 | 0.330 | 8.124 | 0 |
| SS1C1 | 0.274 | 6.739 | 0 |
| SS1C2 | 0.444 | 10.941 | 0 |
| SS1C3 | 0.210 | 5.182 | 0 |
| SS1C4 | 0.330 | 8.124 | 0 |
| SS1D1 | 0.636 | 45.660 | 0 |
| SS1D2 | 0.697 | 17.168 | 0 |
| SS1D3 | 0.551 | 13.555 | 0 |
| VE1 | 0.655 | 16.117 | 0 |
| VE2 | 0.686 | 16.877 | 0 |
| VE3 | 0.483 | 11.888 | 0 |
| VE4 | 0.597 | 14.688 | 0 |
